# Shifting seas and first feeds: gut microbiome dynamics in juvenile chum salmon (*Oncorhynchus keta*) and their climate vulnerability

**DOI:** 10.1016/j.crmicr.2025.100452

**Published:** 2025-07-30

**Authors:** Subrata Kumar Ghosh, Marty Kwok-Shing Wong, Susumu Hyodo, Koji Hamasaki

**Affiliations:** a Atmosphere and Ocean Research Institute, The University of Tokyo, 5-1-5 Kashiwanoha, Kashiwa, Chiba 277-8564, Japan; b Faculty of Fisheries, Chattogram Veterinary and Animal Sciences University, Chattogram 4225, Bangladesh; c Collaborative Research Institute for Innovative Microbiology, The University of Tokyo, 113-8657 Bunkyo-ku, Japan; d Department of Integrated Biosciences, Graduate School of Frontier Sciences, The University of Tokyo, Chiba 277-0882, Japan

**Keywords:** Gut microbiome, Climate change, Fish, Marine heatwave, Development, Chum salmon, Seawater transition, Bacteria, 16S rRNA gene

## Abstract

**Background:**

The early development of host-associated microbiota is crucial for host health and resilience. In migratory fish like chum salmon, the transition from freshwater to seawater and the onset of feeding are critical life stages that may be susceptible to environmental stressors, including those related to climate change. However, the relative influence of diet versus environment on microbiome stabilization remains unclear under ecologically relevant conditions. This study investigated the dynamics of gut bacterial communities in juvenile chum salmon during the first 90 days post-hatching, exploring how first feeding and freshwater-to-seawater transition shape the gut microbiota, with implications for climate sensitivity.

**Results:**

High-throughput 16S rRNA gene amplicon sequencing revealed highly dynamic gut/body microbiota. Prior to feeding, bacterial communities exhibited considerable fluctuations and lacked compositional stability. However, following the onset of feeding at 35 days post-hatching (DPH), the gut microbiota rapidly stabilized, indicating a strong influence of dietary microbes. Dominant taxa in freshwater stages included *Aeromonas, Vibrio, Acinetobacter*, and members of the *Rhizobiaceae* family. Upon transfer to seawater, the microbial community underwent a significant shift, with *Aliivibrio* and *Colwellia* emerging as dominant taxa, alongside the sustained presence of core taxa like *Bartonella* and *Enterococcus*. Interestingly, while many of the stabilized gut taxa post-feeding were derived from the feed, the microbial composition in the gut differed significantly from that of the feed itself, highlighting strong host-mediated selection. This selective filtering was further evidenced by the consistent presence of core bacterial taxa, including *Bartonella, Enterococcus*, and *Acinetobacter* across both freshwater and seawater environments, suggesting these microbes potentially provide essential functions to the host.

**Conclusions:**

This study demonstrates that the gut microbiota in juvenile chum salmon is assembled by both environmental microbial input from diet and water, and is ultimately filtered by the host. This selective process highlights potential vulnerabilities of chum salmon to shifts in feed and ambient microbial communities, both of which are sensitive to environmental changes. Our findings underscore the importance of understanding host-microbe-environment triads for predicting climate-related vulnerabilities in wild salmon and for guiding future microbiome-based aquaculture strategies.

## Highlights

• Initial instability of juvenile chum salmon gut microbiota stabilized after feeding begins.
• Diet, then seawater, drives major gut microbiome shifts.
• The host actively selects core gut microbes across environments.
• Links early microbiome dynamics to climate change vulnerability.

## Graphic abstract

**Figure:**
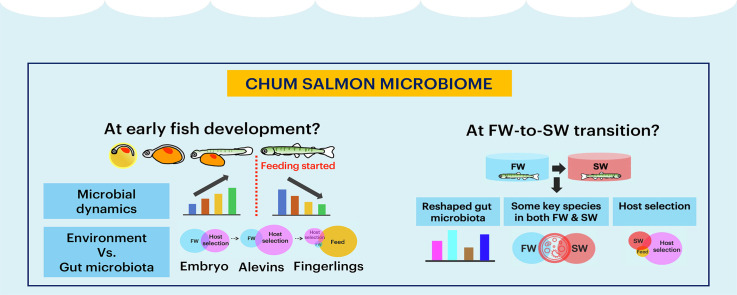

## Introduction

The intricate relationship between animals and their associated microbial communities is increasingly recognized as a critical factor influencing host physiology, development, and resilience to environmental change (Lokesh et al., 2017 Stephens et al., 2016; Avella et al., 2012; Mach et al., 2015; Sommer and Bäckhed, 2013; Ye et al., 2014). In migratory fish species like chum salmon (*Oncorhynchus keta*), the early life stages are particularly vulnerable to environmental stressors. Notably, global climate change, manifested as rising water temperatures and increased frequency of marine heatwaves, poses a significant threat to salmon resources. This impact is particularly severe on juvenile survival during their critical transition from freshwater to marine environments (Kaeriyama and Sakaguchi, 2023). These altered thermal regimes can directly affect salmon physiology and habitat suitability, but also indirectly influence their feeding ecology and the surrounding microbial environment.

The gut microbiota, a key component of the host's physiological machinery, plays a vital role in nutrient acquisition, immune system development, and overall health (Egerton et al., 2018). The establishment and dynamics of this microbial community during early development are influenced by a multitude of factors, including host genetics, diet, and the surrounding environment (Stephens et al., 2016; Bates et al., 2006; Semova et al., 2012; Sommer and Bäckhed, 2013). In salmonids, studies have shown that gut microbial assemblages are species-specific and change with age, habitat, and dietary shifts, such as the transition from live feeds to commercial pellets (Lokesh et al., 2017; Bledsoe et al., 2016; Bakke et al., 2015).

Of particular concern in the context of climate change is the potential disruption of gut microbiota development in juvenile salmon due to altered feeding conditions and the abrupt shift to the seawater microbial environment. Japan's chum salmon populations, especially those returning to the Sanriku coast, have experienced a dramatic decline in recent years, with rising water temperatures implicated as a major contributing factor (Kaeriyama and Sakaguchi, 2023). The high water temperatures in coastal areas are thought to negatively impact juvenile salmon that migrate from rivers to the sea and grow in these nearshore environments. Understanding the influence of environmental factors like water temperature and the feeding environment on the early growth of salmon is therefore crucial.

While the importance of the gut microbiota in fish health is increasingly recognized, research on chum salmon (*O. keta*) remains limited, with our previous work (Ghosh et al., 2022) being a notable exception, and studies focusing on the critical juvenile stage are particularly scarce. This study aims to address this gap by meticulously examining the dynamics of the gut microbiota in captive juvenile chum salmon from before the onset of feeding through their transition to seawater. We hypothesize that the initial dietary intake and the subsequent exposure to the distinct microbial milieu of the seawater environment induce significant and potentially vulnerable shifts in the developing gut microbiota of juvenile chum salmon.

Our experimental design mimics the natural life cycle of chum salmon, encompassing embryonic development, the initiation of exogenous feeding, and salinity acclimation. By analyzing the gut microbial communities at key developmental stages, we aim to: (i) characterize the temporal changes in microbial diversity and community structure, (ii) elucidate the influence of diet on the colonization of gut microbiota, and (iii) determine the impact of the freshwater-to-seawater transition on the gut microbi al composition. Crucially, our findings will provide novel insights into how environmental changes, such as those induced by climate change, might affect the early development and survival of this ecologically and economically important species through the lens of their gut microbiome. Unlike many salmonids that spend extended periods in freshwater, the early seawater migration of chum salmon makes them a particularly valuable model for understanding the rapid influence of the marine environment on gut microbial assembly.

## Materials and methods

### Ethics statement

Chum salmon used in this study were provided by the Iwate Fisheries and Technology Center, Iwate Prefecture, Japan, in January 2022. Fish were humanely euthanized in a buffered solution of 0.1 % 200 mgL^-1^ tricaine methanesulfonate (MS-222; Western Chemical Inc., Furndale, WA, USA). All animal studies were performed according to the Guideline for Care and Use of Animals approved by the Animal Experiment Committee of The University of Tokyo. The present study was carried out in compliance with the Animal Research: Reporting of In Vivo Experiments (ARRIVE) guidelines.

### Fish rearing and experimental design

The fish husbandry and the sampling were performed in the indoor aquarium facility of the Atmosphere and Ocean Research Institute (AORI), The University of Tokyo. Fertilized eggs were collected from the Otsuchi Hatchery, Iwate Prefecture, Northern Japan, from a single artificial fertilization in December 2021. After developing into the eye stage, in early January 2022, 800 embryos were transported to AORI. Embryos were placed in a perforated plastic tray in an indoor 250 L rectangular recirculating tank with continuously aerated freshwater (∼12 °C, pH ∼ 7.5, dissolved oxygen ∼7 mg L^-1^). The temperature of the rearing tanks was regulated by an automatic heating and cooling system. As dissolved oxygen (DO) is a vital factor for fish survival, continuous aeration was provided in each tank to ensure the optimum dissolved oxygen content. About 30 % of the tank water was replaced daily to maintain a relatively clean environment. Upon hatching, approximately 500 sac-fry larvae were randomly collected as our study population. After 28 days post-hatching (DPH), fish were fed once daily to apparent satiation using commercial pellets designed for salmonids: KAIO-3 until 60 DPH and KAIO-4 for the later stages, which were purchased from Marubeni Nisshin Feed Company, Chita City, Aichi Prefecture, Japan. Over the 90-day experimental period, two pellet sizes of the same diet composition (Table 1) were used for feeding the chum salmon. The change in pellet size was to meet the increasing mouth size of the growing fish. To evaluate the changes in the gut microbiota of chum salmon at different developmental stages, fish individuals were sampled at the eye-embryo stage, 3, 7, 10, 14, 21, 28, 35, 42, 50, 60, 75, and 90 days post-hatching (DPH). At 36 DPH, the chum salmon has absorbed their yolk and started feeding. Naturally, these individuals will perform downstream migration to seawater environments as they are considered smolt according to their salinity tolerance (Wong et al., 2019). At this stage, therefore, 100 individuals were transferred to a 250 L round seawater (SW) treatment tank and another 100 individuals to a 250 L round freshwater (FW) control transfer tank. No fish mortality was observed following the transfer. Fish individuals in the SW tank were sampled after 1 day (37 DPH), 7 days (44 DPH), 14 days (51 DPH), 21 days (58 DPH), and 28 days (65 DPH) after the transfer. All fish were fed the same diet described above. The experimental chum salmon were full-sib individuals raised in a communal tank so that the potential influence of host genetics and different environments on the microbiota was reduced.

**Table 1:** Proximate composition of diets fed to chum salmon throughout the study. Two different pellet sizes were administered with the constant diet formulations. KAIO-3 was administered to the very young stages of fish, starting from the exogenous first feeding to 60 DPH, whereas KAIO-4 was designated for the later stages of fish.

Diet name	Pellet Size (mm)	Crude Protein (Min.)	Crude fat (Min.)	Crude fiber (Max.)	Crude ash (Max.)	Crude calcium (Min.)	Crude phosphorus (Min.)
*KAIO-3*	0.3–0.5	≥51.0 %	≥8.0 %	≤3.0 %	≤16.0 %	≥2.0 %	≥1.5 %
*KAIO-4*	0.5–0.7	≥51.0 %	≥8.0 %	≤3.0 %	≤16.0 %	≥2.0 %	≥1.5 %

**Fig. 1:**
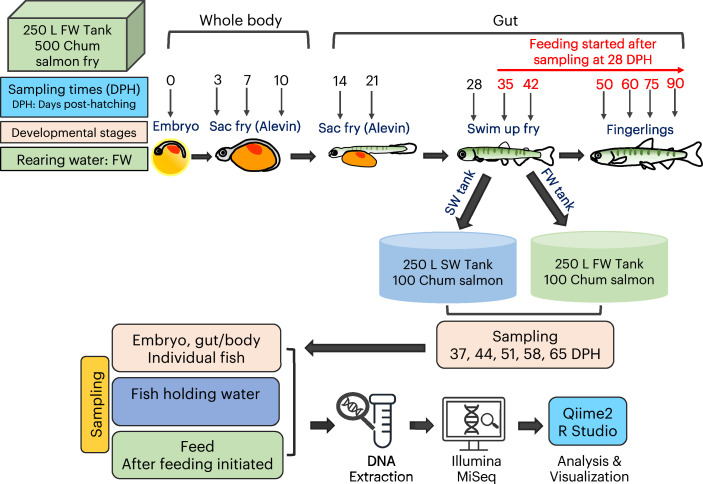
Schematic illustration of the experimental design, depicting the successive developmental stages of chum salmon and husbandry events targeted during the study. The developmental stages of fish are denoted as days post-hatching (DPH). Exogenous feeding commenced after 28 DPH, coinciding with the absorption of the yolk sac. Sampling of gut/body, feed, and holding water was conducted in two conditions: freshwater (FW) from the embryo to 90 DPH and subsequent transfer to seawater (SW) on 37, 44, 51, 58, and 65 DPH. Each sampling occasion involved the collection of 12 fish samples, 6 liters of tank water, and a single diet, which were subsequently processed individually.

A schematic overview of the experimental design and sampling schedule is shown in Fig. 1. At each time point, twelve individuals were sampled and processed individually. The individuals at the embryonic stage, 3, 7, and 10 DPH, were euthanized with MS-222 as described before. Following euthanasia, length and weight were recorded. Because these fish individuals were too small to isolate the intestinal tract, the whole individual was sampled. To ensure only internal microbiota was extracted, a treatment was performed to remove external microbes from euthanized embryos and sac fries, which consisted of submerging the euthanized embryo and larvae in 100 % molecular grade alcohol (FUJIFILM Wako Pure Chemical Corporation, USA) for thirty seconds followed by a brief rinse with laboratory grade ultra-pure water. Immediately following the rinse, those individuals were placed directly into sterile microcentrifuge tubes and stored at −80 °C until further processing.

At all other time points, fish individuals were large enough for dissecting the intestinal tract under sterile conditions. At 14, 21, 28, 35, 37, 42, 44, 50, 51, 58, 60, 65, 75, and 90 DPH, fish were euthanized by MS-222 as described above for five minutes or until cessation of opercular movement (AVMA, 2007). During the feeding period at later stages (35, 37, 42, 44, 50, 51, 58, 60, 65, 75, and 90 DPH), the fish were sampled 20–24 h after feeding to ensure gut evacuation. Following euthanasia, length and weight were recorded. The ventral surface of the fish was washed with 70 % molecular-grade ethanol, and an incision was made from the pectoral fin to the cloaca. Using sterile procedures, the entire intestinal tract and its contents were removed. Samples were placed individually in sterile tubes and stored at −80 °C until further processing. Microbial samples were also collected from the holding tank water and the feeds at all the sampling points to evaluate the differential influence of the environmental and dietary microbes on the intestinal microbiota. Three liters of tank water and a single feed were taken during the representative sampling occasion. Water samples were filtered using 0.22 μm membranes (Sterivex-GP Pressure Filter Unit, Millipore, Merck, Germany) to collect the microbial community in the rearing water. All the feeds and Sterivex filters were stored in sterile tubes at −80 °C until further analysis.

### DNA extraction and PCR amplification of the bacterial 16S rRNA gene

DNA extraction of the fish and feed samples was performed using the Isoil DNA extraction kit (Nippon Gene Co. Ltd., Japan) following the manufacturer’s instructions, with some modifications. Briefly, samples were homogenized into 450 μL Buffer HE and 450 μL enzymatic lysis buffer containing 50 μL of lysozyme (20mg/ml) and incubated at 37 °C for 30 min. Afterward, 50 μL of proteinase K (20 mg/ml) was added, vortexed, and incubated at 55 °C for 30 min. The product was centrifuged at 12,000 × *g* for 15 min before continuing with the standard protocol. DNA extraction of the 0.22 μm membranes from fish-holding water samples was performed using the DNAeasy Blood and Tissue kit following the protocol described by Wong et al. (2020). The DNA concentration was measured with Qubit Fluorometer® using the Qubit dsDNA assay kit (ThermoFisher Scientific Co., USA), and DNA quality was assessed using a Nanodrop spectrophotometer following the manufacturer’s protocol. All the extracted DNA samples were kept at –30 °C until amplification. The sequences of 16S rRNA genes were amplified using the universal bacterial primers 515F (5′-GTGCCAGCMGCCGCGGTAA-3′) and 806R (5′ - GGACTACHVGGGTWTCTAAT-3′) targeting the V4 hypervariable region. V4 regions were chosen for sequencing because they are capable of detecting both bacterial and archaeal taxons with high resolution and show few biases (Wear et al., 2018). Takara Probe qPCR ReadyMix kit (Takara Bio Inc., USA) was used for PCR amplification following the manufacturer’s protocol. Each reaction tube contained 12.5 μL of Takara Probe qPCR ReadyMix and 1 μL (5μM/L) of each primer. Volume was completed to 25 μL with PCR-grade microbial DNA-free water and 1 μL (1 ng/μL) template DNA. PCR was performed in triplicates to reduce PCR bias with the following cycle conditions: initial denaturation at 94 °C for 3 min, followed by 28 cycles of 94 °C for 30 s, 57 °C for 45 s, 72 °C for 1 min, and concluded by a final extension at 72 °C for 5 min. The selection of 28 cycles for V4 region amplification was based on preliminary optimization experiments, which determined this cycle number to be sufficient for robust amplification across all sample types while minimizing the risk of PCR artifacts such as chimeras and heteroduplex formation, as assessed by gel electrophoresis. Electrophoresis on a 1.5 % agarose gel was performed to check the quality of the amplicon DNA. After PCR, the 3 reaction replicates from each sample were pooled in a 0.5 mL PCR tube and kept in a –30 °C freezer until further analysis.

### Library construction and Illumina amplicon sequencing

The amplified DNA was purified by AMPure XP beads (Beckman Coulter Genomics, Brea, CA, United States) to remove primers, dimers, proteins, and phenol. The quality of the resulting DNA was checked by electrophoresis on a 1.5 % agarose gel. The Nextera ® XT Index Kit v2 (Illumina, San Diego, CA, United States) Set A (96 indexes, 384 samples) and Set B (96 indexes, 384 samples) were used for library preparation. Index PCR was performed using KAPA HiFi HotStart ReadyMix® (Roche, USA) according to the manufacturer’s instructions. Index PCR was performed by combining 12.5 μL of 2X KAPA HiFi HotStart ReadyMix® (Roche, USA) and 2.5 μL of each index 1, index 2, and 2.5 μL purified PCR product per sample. Nuclease-free PCR-grade water was added to the final reaction volume of 25 μL per sample. Amplification started with an initial denaturing step for 3 min at 95 °C, followed by 8 cycles of denaturation for 30 s at 95 °C, annealing for 30 s at 55 °C, and extension for 30 s at 72 °C before a final extension for 5 min at 72 °C. The PCR product was then purified using AMPure Beads following Illumina’s 16S Metagenomic Sequencing Library Preparation Protocol (Illumina, Part 15044223, Rev. B). 28 μL of AMPure XP beads was added to each sample. Samples were mixed manually by gently pipetting the entire volume up and down 10 times and incubated at room temperature without shaking. Using a magnetic stand, beads were concentrated on the side of the Eppendorf, and the supernatant was removed. Beads were washed twice with freshly prepared 200 μL ethanol at 80 % and incubated for 30 s at room temperature during each wash. The supernatant was removed, and beads were left to air-dry for 10 min. Tubes were removed from the magnetic stand, and 13.75 μL of 10 mM Tris–HCl pH 8.5 was added for elution. All samples were then quantified by Qubit Fluorometer® using the Qubit dsDNA assay kit (ThermoFisher Scientific Co., USA) following the manufacturer’s instructions. PCR samples were pooled at equimolar concentrations of 4 nM. The quality of the pooled samples was verified on an Agilent 2200 TapeStation® system with High Sensitivity D1000 reagents, followed by heat denaturation of the library, the addition of 10 % PhiX, and dilution to 4 pM. The library was finally run on a single MiSeq flow cell using an Illumina ® MiSeq Reagent Kit v3 (Illumina, San Diego, CA, United States) (600 cycles; 2 × 300) (Catalog Number: MS3203973-600V3). All procedures followed the Illumina guide (16S Metagenomic Sequencing Library Preparation, Part 15044223, Rev. B).

### Sequence data processing, bioinformatics, and statistics

The QIIME2 program (ver. 2023.2.0; Bolyen et al., 2019) was used to analyze the community profile of the reads. The Cutadapt plug-in Martin (2011) was used to eliminate primer sequences. The DADA2 plug-in Callahan et al. (2016) was used to denoise and cluster the reads based on sequence variants (SVs) at a single-nucleotide resolution. The resulting SVs were searched against the SILVA database (Quast et al., 2013). After quality filtering and discarding chimera, chloroplast, mitochondrial, and unassigned sequences, 8199,026 high-quality sequences were retained for analysis. The average number of reads was 66,658 per sample (min=11,890; max=149,414), and 6517 ASVs were obtained. In the estimation of diversity, the number of sequences per sample was rarefied at 11,890 sequence reads. Rarefaction curves were obtained with the number of normalized ASVs. Microbial community structure, diversity, and statistical analyses were performed by processing the normalized (rarefied) ASV table. Rarefaction curves were generated based on observed amplicon sequence variants (ASVs) and (B) Shannon diversity index for each sample (Supplementary Figure S1). Alpha diversity metrics, such as the Shannon-Weiner diversity, Observed ASVs, and Simpson diversity indices, were performed by processing the ASV table in the R environment (R Core Team, v. 4.2.3, 2022) with the Vegan (Oksanen et al., 2018, Oksanen et al., 2020), Phyloseq (McMurdie and Holmes, 2013), Microbiome (Lahti and Shetty, 2012–2019), and ggplot2 (Wickham, 2016) packages. Statistical analysis of alpha diversity indices across samples was conducted in R by one-way ANOVA with a Tukey’s HSD post-hoc test (Supplementary Table 2A). Prior to ANOVA, assumptions of normality were assessed using the Shapiro-Wilk test, and homogeneity of variances was evaluated using Levene's test. Linear discriminant analysis (LDA) effect size (LEfSe) (Segata et al., 2011) was created to identify significantly abundant taxa in each developmental stage of fish according to the web-based tool described by Chong et al. (2020). For LEfSe, the Kruskal–Wallis test by rank was performed to detect the taxa with a significant abundance (*p* < 0.05), followed by LDA to evaluate the effect size of each differentially abundant taxa (LDA cut-off 3.0). The nonmetric multidimensional scaling (NMDS) ordination plot was generated using the normalized (rarefied) ASV abundance data based on Bray-Curtis distances for each time point, including the fish samples, representative rearing water samples, and all diets unique to that time point, and a suitable number of axes was used to reduce stress. A PERMANOVA statistical test was employed to analyze the beta diversity across time (fixed factor of fish age) using 9999 permutations of data, as recommended by Clarke and Gorley (2006) (Supplementary Table 2B). A heatmap depicting the core bacterial taxa in the gut microbiota of chum salmon shared between the freshwater and seawater environments at the ASV level, based on an abundance threshold (> 0.01 %) for taxa with a prevalence greater than 0.9, was generated using MicrobiomeAnalyst 2.0 (Chong et al., 2020).

ASV presence in any given sample type (gut, water, or feed) was determined based on a count of at least one (ASV count ≥ 1) in at least one sample within that category. Overlap (shared ASVs) and uniqueness were calculated using set-based operations (intersections and set differences) on the ASV lists for each environment. To identify the differentially abundant taxa across the chum salmon ontogeny that potentially drive the difference in beta diversity, similarity percentage analysis (SIMPER) was conducted using the Primer software (ver. 7). Before this analysis, ASVs had to be detected in three or more separate fish samples, to remove rare taxa, before conducting the SIMPER analysis on a Bray-Curtis similarity matrix. R software v. 4.2.3 (R core team) and the vegan package (Oksanen et al., 2018) were used for these statistical analyses.

## Results

### Environmental variables and fish growth

The chemistry profile of the holding water is provided in Supplementary Table 1. The water temperature ranged from 12 to 12.3 °C, with salinity in the transfer tank from 34–35 PSU, whereas the pH ranged from 6.7 to 6.9. No fish health issues were evident throughout the experiment. The fork lengths and weights of the fish ranged from (mean ± SD) 2.3 ± 0.012 cm and 0.23 ± 0.01 g at 3 DPH, to 7.3 ± 0.14 cm and 2.3 ± 0.14 g at 90 DPH, respectively (Supplementary Figure S2).

### Diversity and taxonomic profiles of the chum salmon microbiome at different developmental stages

**Fig. 2:**
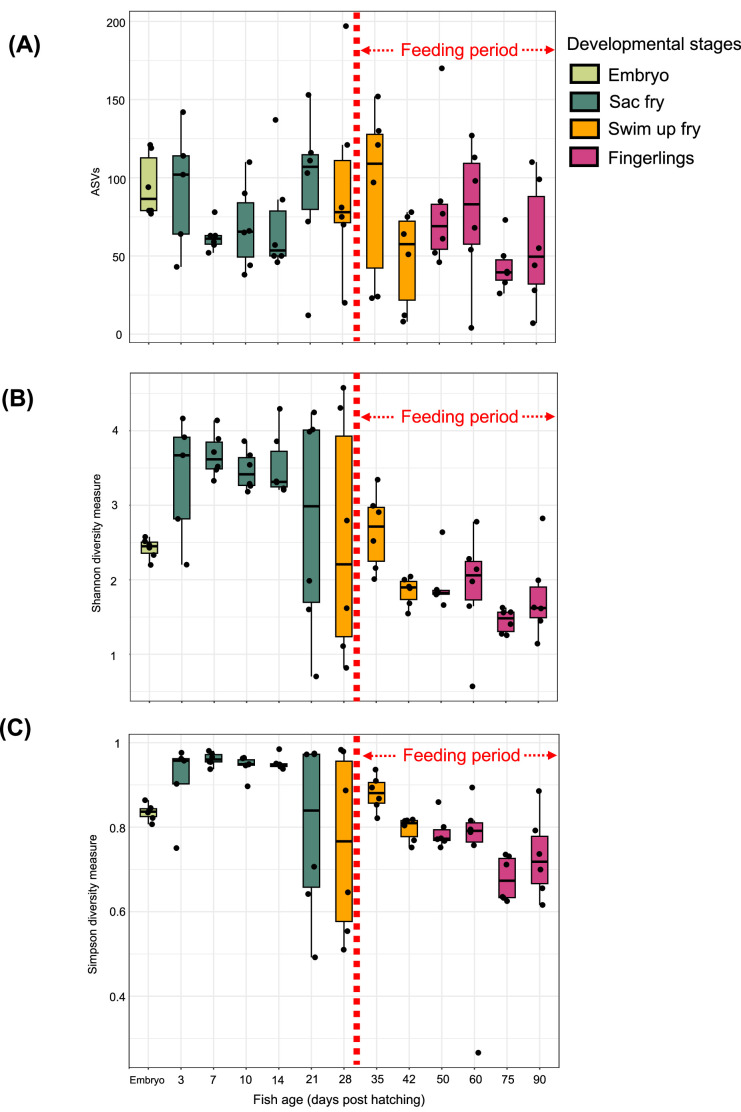
Alpha diversity of chum salmon microbiota during developmental stages. Alpha diversity is indicated by (A) observed ASVs, (B) Shannon, and (C) Simpson diversity indices at different developmental stages of fish. In the estimation of diversity, the number of sequences per sample was rarefied at 11,890 sequence reads, and plots were achieved by processing the ASV table in the R environment (v. 4.2.3, 2022; R Core Team). Statistical analysis of alpha diversity indices across samples was conducted in R by one-way ANOVA with a Tukey’s HSD post-hoc test. Prior to ANOVA, assumptions of normality were assessed using the Shapiro-Wilk test, and homogeneity of variances was evaluated using Levene's test. Each color represents a specific developmental stage of chum salmon expressed as days post-hatching (DPH).

**Fig. 3:**
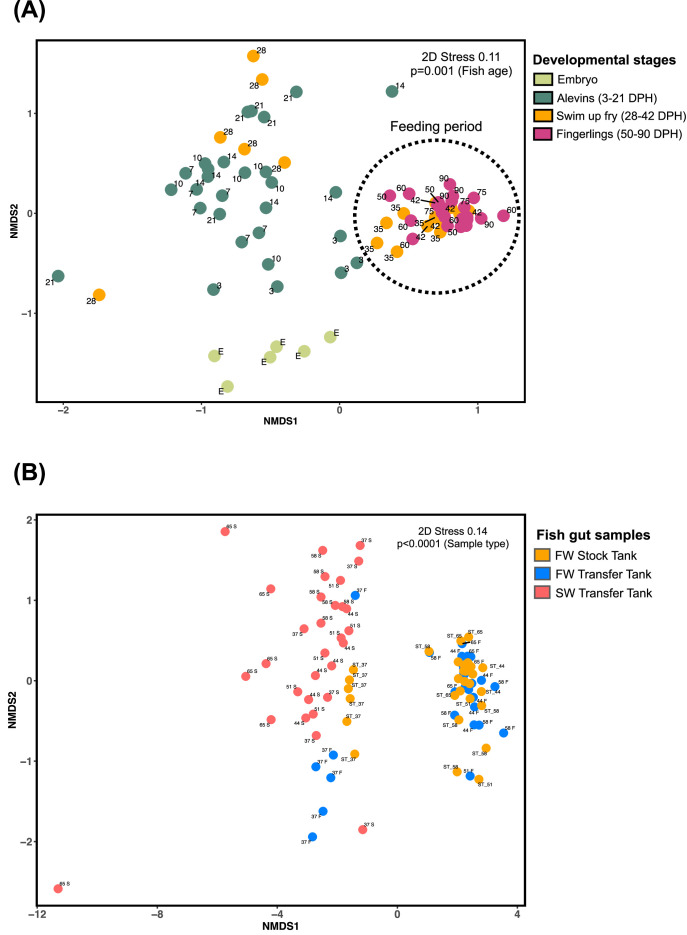
**(A)** Nonmetric multidimensional scaling (NMDS) ordination plot based on Bray–Curtis distance indicating bacterial community dissimilarity at different developmental stages of fish. Each developmental stage is color-coded according to the specific developmental stage of chum salmon expressed as days post-hatching (DPH). The number beside the plot represents the different ages of the fish. “E” represents embryo. **(B)** NMDS ordination plot based on Bray-Curtis distance indicating chum salmon gut bacterial community dissimilarity at different stages in freshwater and seawater. The ordination plot shows the clustering of bacterial communities according to the fish habitat. Each sample plot is color-coded according to the habitat (yellow fish in the freshwater stock tank, blue= fish in the freshwater transfer tank, red= the seawater transfer tank counterpart). The number beside the plot represents different ages of fish as shown as days post-hatching (DPH), F=freshwater, S=seawater, and ST=stock tank. A PERMANOVA statistical test was employed to analyze the beta diversity across time (fixed factor of fish age for figure A, fixed factor gut sample type, i.e., freshwater or seawater) using 9999 permutations of data.

The average ASVs detected in each cohort of the chum salmon microbiome ranged from 48 to 130, with some extent of individual differences (Fig. 2A; Supplementary Table 4). The Alpha diversity indices, including Shannon-Weiner and Simpson species richness and evenness indices, showed higher gut bacterial community diversity at the yolk-sac stage. Diversity remained stable between 28 and 35 DPH, but decreased from 42 DPH onwards (Fig. 2). The beta diversity shown by non-metric multidimensional scaling (NMDS) suggested clustering of community structure by fish age or developmental stages before the start of feeding (Fig. 3A). Microbiome at the embryonic stage formed a significantly distinct cluster. In contrast, alevin samples from 3 DPH to 21 DPH formed a moderate cluster, with some overlapping with 28 DPH swim-up fry samples. However, the overlapping was significantly prominent starting from 35 DPH to 90 DPH, when feeding started. Statistical analyses of the alpha (Shannon-Weiner) diversity and the beta diversity from different developmental stages suggested a significant stage-dependent difference (PERMANOVA, *p* < 0.001, 9999 permutations, fixed factor of fish age) of the microbial consortia. Furthermore, pairwise PERMANOVA results (Supplementary Table 2C) suggested that significant shifts occurred in bacterial beta diversity between different age groups of chum salmon.

**Fig. 4:**
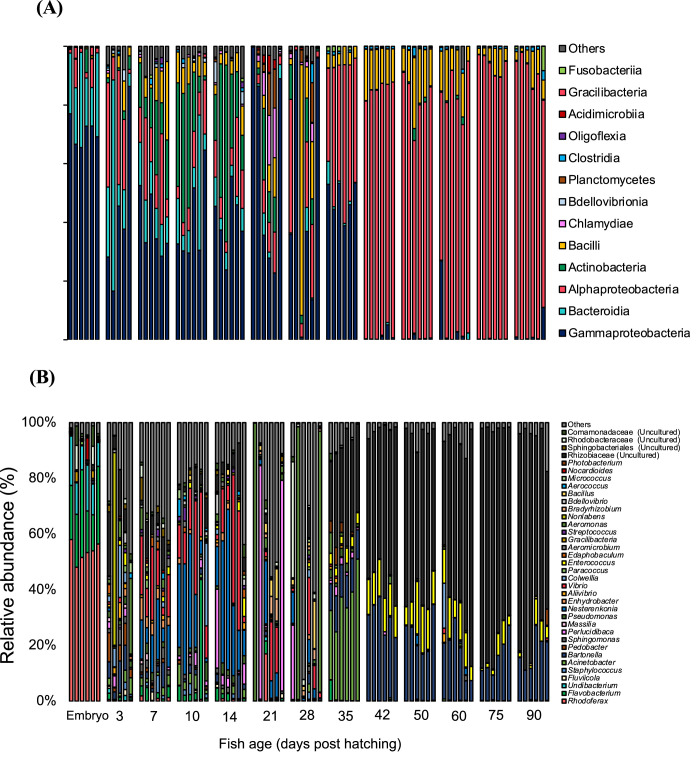
Bacterial community composition of chum salmon at different developmental stages in freshwater, shown at the (A) class level and (B) genus level. Bacterial relative abundance data are shown as the value calculated from six replicates of each sample. Bacterial classes and genera that contributed less than 2 % were combined and referred to as “others”. Bacterial taxa not classified up to the genus level are displayed at the highest taxonomic level available.

The chum salmon microbiome at different stages of development had a diverse community represented by ten phyla with more than 0.1 % relative abundance. Among them, *Proteobacteria, Bacteroidota, Firmicutes*, and *Actinobacteriota* were the most abundant (Supplementary Figure S3). At the class level, *Gammaproteobacteria* was the most abundant taxa followed by *Alphaproteobacteria, Bacteroidia, Acinetobacteria*, and *Bacilli* (Fig. 4A). At the genus level, there was a clear shift in the microbial communities at different developmental stages. The microbial communities of the embryos were composed mainly of *Rhodoferax* (53.6 ± 3.5 %), *Flavobacterium* (20.9 ± 7.3 %), and *Undibacterium* (12.3 ± 3.9 %) (Fig. 4B). Following hatching, the bacterial community shifted, where genus *Aeromonas* (7.3 ± 5.3 %), *Colwellia* (6.3 ± 3.4 %), and *Pedobacter* (5.7 ± 3.6 %) became major groups at 3 DPH with difference between individual fish samples. At 7 DPH, the dominant bacterial communities shifted to the genus *Nesterenkonia* (17.7 ± 6.4 %) and *Vibrio* (16.5 ± 8.2 %), and their relative abundance was maintained at a high level until 28 DPH. Concurrently with the initial feeding at 35 DPH, significant increases in the relative abundance of the genus *Acinetobacter* (34.9 ± 9.9 %), *Bartonella* (11.4 ± 1.2 %), and *Enterococcus* (3.8 ± 1 %) were observed. Notably, the latter two genera, namely *Bartonella* and *Enterococcus*, consistently maintained a high relative abundance within the fish gut bacterial communities from 35 DPH to 90 DPH. From 35 DPH onwards, there was a clear spike in the relative abundance of an unclassified genus of the Rhizobiaceae family, and it continued to grow in the gut of developing chum salmon (Fig. 4B) during the later stages of the study period.

**Fig. 5:**
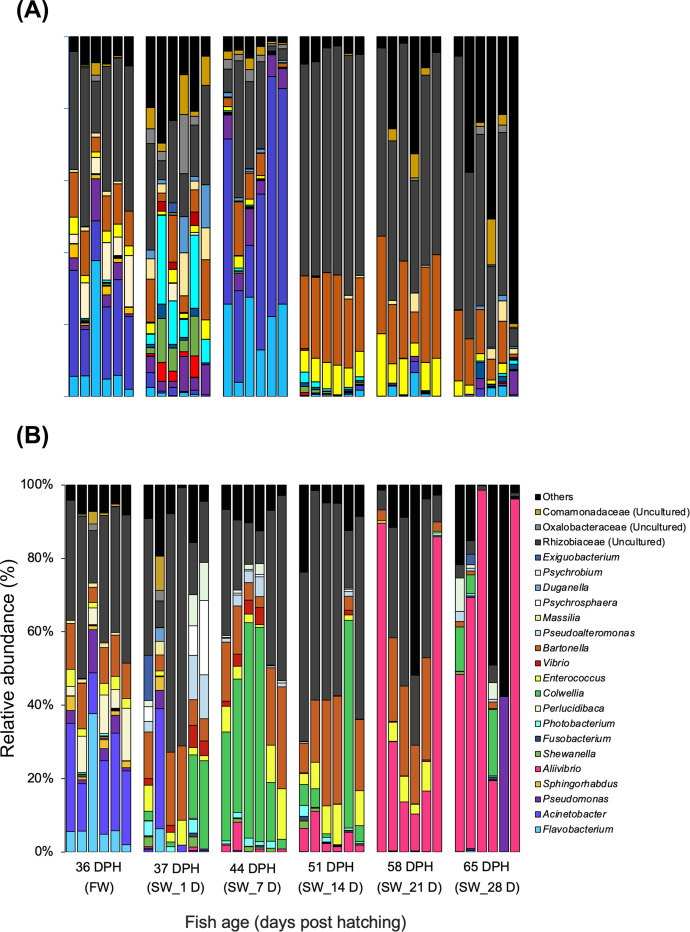
Bacterial community composition of chum salmon at different developmental stages during seawater transition, shown at the genus level, where (A) gut microbial community in freshwater transfer tank, and (B) major taxa in the seawater transfer tank. Bacterial relative abundance data are shown as the value calculated from six replicates of each sample. Bacterial taxa not classified up to the genus level are displayed at the highest taxonomic level available.

On the other hand, during the salinity challenge experiment, the bacterial communities in the gut did not significantly change in the control FW-to-FW transfer (Fig. 5A). When the fish was transferred from FW to SW, the gut microbial communities changed significantly, which was indicated by the non-metric multidimensional scaling (NMDS) plot showing that the bacterial communities in both the stock FW group and FW control transfer group clustered together while those in the SW transfer group formed a distinct cluster (Fig. 3B). Upon SW transfer on 37 DPH, the gut microbial communities shifted to *Bartonella* (11.7 ± 6.1 %), *Colwellia* (7.6 ± 4.6 %), *Enterococcus* (3.6 ± 2 %) and the unclassified genus of Rhizobiaceae family (35.1 ± 21.2 %) (Fig. 5B). After 7 days transfer to SW (44 DPH), the relative abundance of *Colwellia* (33.6 ± 19.1 %) increased in the gut microbial communities. However, the most notable gut microbial community shift was the increase in the relative abundance of *Aliivibrio* after SW transfer (4.7 ± 2.3 % at 51 DPH, 41 ± 24 % at 58 DPH, and 55.2 ± 35.3 % at 65 DPH), which became a dominant taxon of the gut microbiota in SW.

Although there was a bacterial shift following the SW transfer, some overlapping of ASVs between FW and SW fish was also evident. To comprehensively characterize the persistently present microbiota across varying conditions, we defined core ASVs based on their prevalence (percentage of samples in which an ASV was detected) across all samples at a minimum relative abundance threshold of 0.01 %. Two ASVs identified as uncultured Rhizobiaceae bacteria and the other 2 ASVs of the genus *Bartonella* (phylum *Proteobacteria*) were commonly present in >90 % prevalence of all samples at the level of 0.01 % relative abundance regardless of salinities. Also, the ASVs of Lactobacillales bacteria and the genus *Enterococcus* (phylum *Firmicutes*) were present in >60 % prevalence of all samples. Some other ASVs, including well-known intestinal microbes such as *Aliivibrio* and *Acinetobacter,* were consistently identified in >30 % prevalence of all samples. This multi-tiered definition of core ASVs provides a robust representation of microbial groups consistently associated with juvenile chum salmon, reflecting varying degrees of host colonization success or selection irrespective of environmental salinity changes. While some studies apply a single high prevalence threshold (e.g., >50 % or >80 %) for core microbiome definition, our tiered approach allowed for a nuanced identification of taxa that persist with different frequencies across highly dynamic conditions, reflecting their potential functional importance to the host.

### Temporal variability in chum salmon microbiome

Linear discriminant analysis (LDA) effect size (LEfSe) was performed to characterize the significantly abundant taxa in each developmental stage of chum salmon (Supplementary Figure S4). The bacterial communities were significantly different at each developmental stage. The major taxa were *Rhodoferax* in the embryonic stage, *Aeromonas* sp. in 3 DPH, *Vibrio* in 7 DPH, *Nesterenkonia* in 10, 14 DPH, *Aeromonas* in 21, 28 DPH, *Acinetobacter* in 35 DPH, *Bartonella* and uncultured Rhizobiaceae (not classified up to the genus level) in 42, 50, 60, 75, 90 DPH. To further explore the microbial dissimilarities over time, SIMPER analysis was performed to evaluate within-group similarity and among-group dissimilarity, after filtering taxa that were not detected in at least three separate gut samples. This analysis detected the microbial taxa that were most typical of individual age groups, as well as those most discriminatory between groups (Supplementary Table 3). The phyla *Proteobacteria* and *Firmicutes* prevailed at all developmental stages, with *Alphaproteobacteria, Gammaproteobacteria*, and *Bacilli* being the dominant classes. At the order level, *Rhizobiales* from *Alphaproteobacteria, Pseudomonadales,* and *Burkholderiales* from *Gammaproteobacteria* were common at all developmental stages. Among-group dissimilarity analysis indicated that the microbiota from the embryo and 28 DPH larvae were the most dissimilar (97.61 %) whereas those of 75 DPH and 90 DPH were the least dissimilar (36.02 %). At the genus level, the most abundant genus at each developmental stage was responsible for the significant differences in the bacterial communities which included *Rhodoferax* (embryo), *Aeromonas* (3 DPH), *Vibrio* (7 DPH), *Nesterenkonia* (10, 14 DPH), *Aeromonas* (21, 28 DPH), *Acinetobacter* (35 DPH), and *Bartonella* (42, 50, 60, 75, 90 DPH). The LEfSe test was also employed to characterize the significantly abundant bacterial taxa during the FW-to-SW transition, resulting in the identification of different communities in both habitats. The most abundant taxa in each phase that was responsible for the significant differences in the bacterial communities include *Enterococcus, Acinetobacter, Bartonella, Flavobacterium* in the FW stock group, *Enterococcus, Photobacterium* and *Bartonella* in FW.1D, *Acinetobacter* and *Flavobacterium* in FW.7D It is noted that the uncultured genus of Rhizobiaceae family, *Bartonella*, and *Enterococcus* prevailed in all of the gut samples in FW. Upon transfer to SW, the significantly abundant taxa in the gut comprised mainly of *Enterococcus, Bartonella, Colwellia*, and *Acinetobacter* in 37 DPH (SW.1D), *Colwellia* in (44 DPH, SW.7D), and *Aliivibrio* in the later stages in SW (51 DPH, SW.14 D; 58 DPH, SW.21D; and 65 DPH, SW.28D).

### Correlations between the gut and environmental microbiota

To quantify the environmental microbial contribution to the gut microbiome, we performed a detailed ASV overlap analysis between gut, water, and feed samples for both freshwater and seawater conditions (Tables 2A and 2B). In the freshwater tank, out of 2618 total unique ASVs in the gut, 458 ASVs were shared with water (representing 17.49 % of gut ASVs), and 140 ASVs were shared with feed (representing 5.35 % of gut ASVs). A small proportion of ASVs (18 ASVs, 0.69 % of gut ASVs) were shared across gut, water, and feed, indicating a limited direct exchange from the overall environment. Notably, a significant proportion of gut ASVs were unique to the gut (2038 ASVs), suggesting host-specific colonization.

**Table 2:** Quantitative summary of ASV sharing and uniqueness between gut, environmental water, and feed. These tables present a quantitative overview of ASV (Amplicon Sequence Variant) sharing and uniqueness among juvenile chum salmon gut, environmental water, and dietary feed during both freshwater (A) and seawater (B) acclimation phases. Data were derived from 16S rRNA gene amplicon sequencing. ASV presence in any given sample type (gut, water, or feed) was determined based on a count of at least one (ASV count ≥ 1) in at least one sample within that category. Overlap (shared ASVs) and uniqueness were calculated using set-based operations (intersections and set differences) on the ASV lists for each environment. Counts represent the absolute number of ASVs, and percentages indicate the proportion of ASVs from the respective category.

A.				
Category	ASVs count	% of gut ASVs	% of water ASVs	% of feed ASVs
Total unique ASVs in gut	2618			
Total unique ASVs in water	3073			
Total unique ASVs in feed	329			
Shared ASVs (gut & water)	458	17.49 %	14.9 %	
Shared ASVs (gut & feed)	140	5.35 %		42.55 %
Shared ASVs (water & feed)	18		0.59 %	5.47 %
Shared ASVs (gut, water & feed)	18	0.69 %	0.59 %	5.47 %
Unique to gut	2038			
Unique to water	2615			
Unique to feed	189			

B.				
Category	ASVs count	% of gut ASVs	% of water ASVs	% of feed ASVs
Total unique ASVs in gut	2633			
Total unique ASVs in water	5420			
Total unique ASVs in feed	619			
Shared ASVs (gut & water)	621	23.59 %	11.46 %	
Shared ASVs (gut & feed)	232	8.81 %		37.48 %
Shared ASVs (water & feed)	34		0.63 %	5.49 %
Shared ASVs (gut, water & feed)	32	1.22 %	0.59 %	5.17 %
Unique to gut	1812			
Unique to water	4797			
Unique to feed	385			

Similarly, after seawater transfer, the gut contained 2633 unique ASVs, with 621 ASVs shared with seawater (23.59 % of gut ASVs) and 232 ASVs shared with feed (8.81 % of gut ASVs). The number of ASVs shared among gut, water, and feed also increased (32 ASVs, 1.22 % of gut ASVs) compared to freshwater, reflecting the dynamic nature of the marine environment. Despite this, 1812 ASVs remained unique to the gut, further supporting the idea of a distinct gut community. These quantitative analyses demonstrate that while environmental and dietary sources contribute to the gut microbiome, a substantial portion of the gut community consists of unique or specifically enriched ASVs.

Samples of chum salmon and corresponding water were collected throughout the study to compare the microbial relationship between the gut and the environment. The total unique number of bacterial ASVs in the body/gut (2618) was higher than that in feed samples (329) but lower than that in holding water (3073). Body/gut samples had lower alpha diversities than water samples, but the alpha diversities of body/gut samples were higher than those of feed samples in all diversity indices (Supplementary Figure S5). Furthermore, PERMANOVA results indicated that the microbial communities derived from body/gut, feeds, and water were significantly different (*p* < 0.001, 9999 permutations, Supplementary Table 2B). Moreover, the microbial community dissimilarity based on the Bray-Curtis distance in the NMDS plot suggested a distinctly separated clustering of the body/gut and water-derived microbiota, whereas the microbial communities in the feed were in close proximity to the gut-derived microbiota with exogenous feeding (Fig. 7).

Microbial communities in the body/gut and tank water were classified at the genus level as shown in Fig. 4B & Supplementary Figure S6 (B), respectively. In embryos, the most abundant ASVs, including *Rhodoferax* (53.6 ± 3.5 % average relative abundance), *Flavobacterium* (20.9 % 20.9 ± 7.3 % average relative abundance), *Undibacterium* (12.3 % 12.3 ± 3.9 % average relative abundance), represented only 0.7 ± 0.3 %, 0.5 % and 0.4 % ± 0.2 %, of ASVs in rearing water, respectively. Similarly, in 3 DPH, the most abundant ASVs, including *Aeromonas* (7.3 ± 5.3 %), *Colwellia* (6.3 ± 3.4 %), and *Pedobacter* (5.7 ± 3.6 %), represented <0.1 % of ASVs in rearing water. After the yolk sac was absorbed and feeding initiated, a large part of the gut microbial communities was shaped by the communities originating from the feeds, such as the genus *Bartonella* and *Enterococcus* (Fig. 4B and Supplementary Figure S6 (A). Some other dominant taxa that overlapped between the gut and feeds included *Photobacterium, Vibrio, Lactobacillus, Shewanella*, and *Fusobacterium*. Upon initial SW transition, there was some overlapping of taxa between the gut and respective water samples, including *Acinetobacter, Perlucidibaca, Massilia, Flavobacterium*, and later from the respective feed samples, such as *Bartonella* and *Enterococcus* (Supplementary Figure S7). However, as the fish acclimated to the SW over time, the influence of environmental microbiota on gut microbial communities started to decline, and the intestinal communities differed significantly from those of the water and feed, as shown by the proportion of abundant ASVs from various sources. For example, the most abundant taxon in the gut of 44 DPH was *Colwellia* (33.6 ± 19.1 %), whereas it only represented 0.5 % in the rearing seawater (Supplementary Figure S7 (B)). Similarly, the relative abundance of *Aliivibrio* in the gut at 58 and 65 DPH was 41 ± 24 % and 55.2 ± 35.3 %, respectively, but represented only 1.5 ± 0.3 % and 4 ± 0.6 %, respectively, in the holding water, and this ASV was absent in the respective feed sample (Supplementary Figure S7).

## Discussion

The gut microbiome plays a pivotal role in maintaining the health and physiological functions of fish (Wu et al., 2012; Bacanu et al., 2013; Llewellyn et al., 2014). Our study provides a comprehensive longitudinal analysis of the gut microbiota dynamics in juvenile chum salmon during key early life stages, revealing significant shifts associated with the onset of feeding and the transition to seawater. Notably, our findings offer a unique perspective on how these fundamental ecological transitions, potentially exacerbated by climate change-induced environmental alterations, can impact the developing gut microbiome of this migratory species.

### Succession of microbiota in the embryonic and alevin stages

The chorion of fish eggs is rich in bacterial substrates (Treasurer et al., 2005; Wedekind et al., 2010, 2002), allowing bacteria to colonize at high abundance at early chorionic stages (Llewellyn et al., 2014; Hansen, 1999). In this study, the microbial community of the chum salmon embryo was dominated by the genera *Rhodoferax, Flavobacterium*, and *Undibacterium*. These bacteria could be specific colonizers on the egg of chum salmon because of the rapid colonization and biofilm formation by both *Rhodoferax* and *Flavobacterium* (Barnes et al., 2009; Wilkins et al., 2015). *Rhodoferax* species are purple non-sulfur bacteria that are frequently found in aquatic systems that are exposed to light, although they are also common in ditchwater and activated sludge (Wilkins et al., 2015). *Undibacterium* was reportedly abundant on the egg surface of Atlantic salmon in freshwater (Lokesh et al., 2019), which is in agreement with our present data.

The bacterial community of chum salmon alevin was highly diverse and significantly different from those of the unhatched embryos, which contained mainly the class *Gammaproteobacteria, Alphaproteobacteria, Actinobacteria, Bacteroidia*, and *Bacilli*. At the genus level, *Nesterenkonia, Vibrio*, and *Aeromonas* were relatively abundant. These significant differences could be due to the exposure of fish skin and gill to the environment upon hatching, in which a different bacterial substrate was provided by the fish mucosal system (Fujimoto et al., 2013; Galindo-Villegas et al., 2012; Zapata et al., 2006). Also, it was reported that salmonids respire through cutaneous breathing before hatching, which will be changed to pharyngeal breathing after hatching, and the shift may subsequently affect the bacterial community (Wells and Pinder, 1996). Alevin is a critical stage as it is the first stage at which the fish is in contact with the microbe-rich environment (Fujimoto et al., 2013; Galindo-Villegas et al., 2012; Zapata et al., 2006), where the host immune system is developing, and adaptive responses could be weak (Zapata et al., 2006). The diverse microbial community among alevin stages might be advantageous for the host to defend against potential pathogens (Liu et al., 2014; Llewellyn et al., 2014; Rawls et al., 2004).

### Succession of gut microbiota after the start of feeding

When the nutrients in the yolk sac are consumed, exogenous feeding is essential. The commencement of exogenous feeding at 35 DPH triggered a significant decrease in gut microbial diversity, accompanied by a notable increase in the relative abundance of *Bartonella* and *Enterococcus*. The presence of these genera in the feed suggests a direct dietary influence on gut microbial composition, although the lower abundance of other feed-associated bacteria like *Photobacterium, Shewanella*, and *Fusobacterium* in the gut hints at potential host selection or differential colonization capabilities (Burgos et al., 2018; Ingerslev et al., 2014; Romero and Navarrete, 2006; Stephens et al., 2016). While some *Bartonella* species are pathogenic in mammals (Breitschwerdt and Kordick, 2000), our findings suggest a potential for commensal colonization in fish. Similarly, while certain *Enterococcus* species can be pathogenic (Alshammari et al., 2019; Zahran et al., 2019), others exhibit beneficial roles, such as promoting mucus production and inhibiting pathogens (Rodriguez-Estrada et al., 2009; Alshammari et al., 2019). This highlights the complex and context-dependent nature of host-microbe interactions in developing fish.

Regarding *Bartonella*, while various species are well-documented as pathogenic in mammalian hosts (Breitschwerdt and Kordick, 2000), our study observed its sustained presence and increased relative abundance in the chum salmon gut without any overt signs of disease or mortality in the fish throughout the experimental period. This observational evidence, in the absence of histopathological findings or specific pathogenicity assays, suggests that the *Bartonella* species encountered in this study may either be non-pathogenic to chum salmon under these conditions or exist in a commensal relationship within the fish gut. Further targeted research, including isolation and characterization of specific *Bartonella* strains from fish, is required to definitively ascertain their symbiotic or pathogenic potential in aquatic hosts.

### Gut microbial shifts following the transition from freshwater to seawater

Why does SW matter? It is because SW fish need to drink SW constantly to compensate for the water loss by osmosis since the SW (∼1000 mOsm) is hyperosmotic to the body fluid (∼300–350 mOsm) of bony fish (Takei, 2021; Grosell, 2010). Bony fishes are capable of desalinating the imbibed SW to absorb the osmotic-free water from the drinking, and the absorbed ions are then excreted by the ionocytes in the gill (Whittamore, 2012). Atlantic salmon smolt drank ∼1.6 % v/w seawater a day, and the drinking increased to 1.9 % v/w with feeding (Usher et al., 1988). Besides adult fish, larval and juvenile fish also need drinking in SW (Mangor-Jensen and Adoff, 1987), and a burst of drinking was evident when the fish were transferred from FW to SW (Kaneko and Hasegawa, 1999). However, FW fish have their body fluid hyperosmotic to the environmental water (∼0–1 mOsm), so they do not need to drink (Perrott et al., 1992). Our experimental scheme mimicked the natural downstream migration of chum salmon by transferring the emerged juveniles from FW to SW. Since drinking is a universal behavior by bony fish in SW, the abrupt transfer of juvenile chum salmon from FW to SW would initiate a burst of drinking that introduced the novel microbiota from the imbibed SW, disrupting the existing communities (Reitan et al., 1998; Olafsen, 2001). Our results suggested that the change in gut microbiota in the SW group was neither a tank effect nor a handling stress effect. The clear impact of drinking SW on gut microbial communities was supported by the statistical analysis of the beta diversity, which indicated that the fish gut microbial consortia between the FW and SW were significantly distinct (PERMANOVA, *p* < 0.0001, 9999 permutations).

The transition from freshwater to seawater induced a dramatic shift in the gut microbial community, with the emergence of *Aliivibrio* as a dominant taxon. This observation aligns with previous studies on salmonids in marine environments (Godoy et al., 2015; Gupta et al., 2019), indicating that *Aliivibrio* is a common inhabitant of the SW-acclimated salmonid gut. Given that seawater ingestion is essential for osmoregulation in marine fish (Takei, 2021; Grosell, 2010; Usher et al., 1988; Perrott et al., 1992), the introduction of novel marine microbes via drinking likely plays a significant role in this community shift. While some *Aliivibrio* species are mutualistic (Dunn, 2012), others are opportunistic pathogens whose virulence can be exacerbated by environmental stressors such as elevated water temperatures (Ghosh et al., 2022; Egerton et al., 2018; Hjerde et al., 2008).

### Host selection and possible roles of core taxa

Significant disparity between the gut and environmental microbiota, when viewed in conjunction with the overall ASV sharing data (Table 2B), strongly suggests active host-mediated selection or preferential colonization of certain marine-derived taxa. The fact that a large percentage of gut ASVs are unique to the gut, despite constant exposure to waterborne microbes, further supports active host-mediated selection of environmental microbiota, establishing a gut community distinct from the ambient water, even as new microbes are constantly introduced via drinking. This active shaping process, quantified by the ASV uniqueness and overlap ratios, is crucial for forming a stable, functional microbiome suited to the marine environment.

**Fig. 6:**
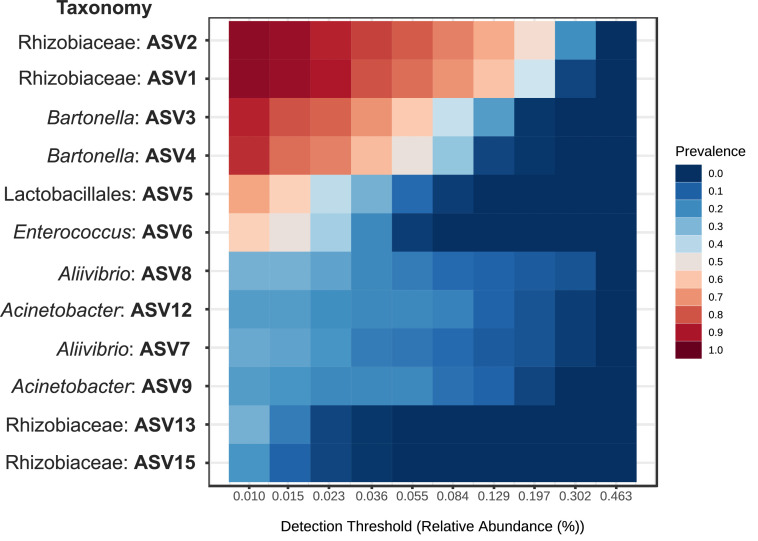
Heatmap showing the core bacterial taxa in the gut microbiota of chum salmon shared between the freshwater and seawater environments at the ASV level, based on an abundance threshold (> 0.01 %) for taxa with a prevalence greater than 0.9. The x-axis represents the detection thresholds, with lower abundance values on the left and higher abundance values on the right. The color shading represents the prevalence of each bacterial genus among the samples for each abundance threshold. As the detection threshold increases, the prevalence decreases.

Despite the significant microbial turnover associated with feeding and seawater transfer, the persistence of core taxa such as Bartonella, Enterococcus, Acinetobacter, and an uncultured genus of Rhizobiaceae across different salinity conditions suggests a degree of host selection for microbes (Fig. 6). This core microbiome may represent a crucial foundation for gut health and resilience in chum salmon, irrespective of the immediate environmental microbial pool.

**Fig. 7:**
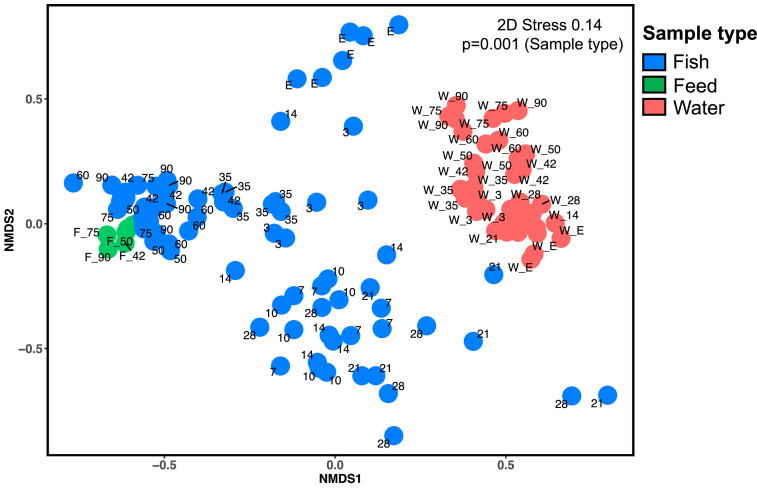
Nonmetric multidimensional scaling (NMDS) ordination plot based on Bray-Curtis distance indicating bacterial community dissimilarity among fish, water, and feed samples during the study period. Ordination plots show the clustering of bacterial communities according to the sample type. Each sample plot is color-coded according to the type (blue= fish, red= water, and green= feed. The number beside the plot represents different ages of fish, as shown in days post-hatching (DPH). “E” represents embryo. A PERMANOVA statistical test was employed to analyze the beta diversity across gut, feed, and water samples (fixed factor of sample type) using 9999 permutations of data.

While the precise mechanisms of selective filtering and enrichment of specific microbial taxa remain to be fully elucidated in chum salmon, existing literature points to several potential underlying biological processes in fish. The host immune system, particularly the mucosal immune responses mediated by specialized cells and immunoglobulin-like molecules, is a critical regulator of gut microbial composition (Lightfoot et al., 2021; Salinas et al., 2011). Fish gut mucus, composed of glycoproteins and mucins, provides a physical barrier and a unique chemical environment that can selectively promote or inhibit microbial adherence and growth (Van der Marel et al., 2010; Bolnick et al., 2014). Specific host receptors, such as Toll-like receptors (TLRs) and nucleotide-binding oligomerization domain (NOD)-like receptors (NLRs), can recognize conserved microbial patterns, triggering tailored immune responses that influence colonization success (Kanwal et al., 2020; Sun et al., 2020). Furthermore, the unique physiological conditions within the fish gut, including pH gradients, oxygen levels, digestive enzyme profiles, and bile acid concentrations, act as powerful selective pressures, favoring the proliferation of microbes adapted to these specific niches while excluding others (Merrifield and Rodiles, 2015; Liu et al., 2016). Therefore, the consistent presence of core taxa like *Bartonella, Enterococcus*, and *Acinetobacter* across varying salinities likely reflects their ability to either evade detrimental host responses or leverage specific host recognition pathways and mucosal patterns that facilitate their stable colonization and potentially beneficial interactions within the chum salmon gut.

The present study documented some key bacterial species in the gut of chum salmon in FW and SW environments, including *Aeromonas, Vibrio, Enterococcus*, Lactobacillales, *Acinetobacter, Bartonella*, and *Aliivibrio*, which were also found in some previous studies (Ghosh et al. 2022; Wu et al., 2012; Egerton et al., 2018). Although some strains of *Aeromonas* and *Vibrio* are known to be pathogenic, the chum salmon in this study exhibited no mortality or signs of disease throughout the experiment. Thus, the key microbiota detected in this study could be neutral or beneficial to the normal functions of the chum salmon gut. Members of the genus *Aeromonas* are normal components of the fish gut microbiota, and they are considered as an autochthonous community in the guts of both FW and SW environments (Roeselers et al., 2011; Wu et al., 2012; Kim et al., 2007; Nayak 2010; Wu et al., 2010), which is beneficial to digestion and immune functions (Ringø, 2003; Ray et al., 2012). Vibrios are diverse and widely distributed taxa in the gut of various fish, including salmonids, in both FW and SW environments (Ghosh et al., 2022; Egerton et al., 2018, Lokesh et al., 2019, Keiz et al., 2023, Ray et al., 2012). The genus is important in aquaculture as many *Vibrio* species can produce hydrolytic enzymes that assist in the breakdown of dietary components. Some species were known to produce cellulase (Itoi et al., 2006; Sugita and Ito, 2006), amylase (Hamid et al., 1979; Gatesoupe et al., 1997), lipase (Gatesoupe et al., 1997; Henderson and Millar, 1998), chitinase (MacDonald et al., 1986), and other digestive enzymes (Ray et al., 2012). For instance, *Vibrio alginolyticus* is used as a probiotic in the culture of Atlantic salmon to decrease *Aeromonas salmonicida* infection (Austin et al., 1995).

It is crucial to acknowledge that many *Aeromonas* and *Vibrio* species exhibit conditional pathogenicity, capable of causing disease outbreaks in fish, particularly when the host is stressed or environmental conditions are suboptimal For instance, elevated water temperatures, a significant concern in the context of climate change, are known to exacerbate the virulence of certain *Vibrio* species and contribute to *Aeromonas*-related infections in aquatic organisms (Ghosh et al. 2022; Wu et al., 2012). Therefore, while these genera may serve beneficial roles in healthy fish, their prevalence in the gut of juvenile chum salmon also highlights a potential vulnerability, where environmental stressors could shift their interaction from commensal to pathogenic, impacting fish health and survival.

### How will this study help the related field?

The findings of this study have significant implications for aquaculture practices, particularly in the context of enhancing the survival of hatchery-reared chum salmon released for sea ranching (Miyakoshi et al., 2013). Our demonstration of the dynamic nature of the gut microbiota during early development and the substantial impact of seawater transfer highlights critical windows for potential probiotic interventions. Specifically, the rapid stabilization of the gut microbiota after initial feeding and the dramatic community restructuring upon seawater introduction (e.g., the emergence of *Aliivibrio* and shifts in diversity) highlight critical windows for microbial intervention strategies. For instance, while the early alevin stage has high microbial plasticity, the data suggest that any microbial amendments (e.g., via feed) might be significantly disrupted or altered once the fish are transferred to seawater, where compulsory drinking introduces novel microbial landscapes. Therefore, our results imply that if probiotic administration is considered to improve fingerling fitness, its timing and duration are crucial. Initial supplementation of probiotics at first feeding might need to be followed by continued or modified administration after seawater transfer to ensure sustained colonization by beneficial microbes adapted to the marine environment. Furthermore, considering the increasing challenges posed by climate change, manipulating the gut microbiota of juvenile salmon through targeted probiotic applications could potentially enhance their resilience to thermal stress and pathogen proliferation in a warming ocean. Our work provides a foundational understanding that can inform future targeted probiotic applications designed to potentially enhance the resilience of juvenile salmon to thermal stress and pathogen proliferation in a warming ocean by supporting the establishment of a robust and adaptive gut microbiome from early life stages.

## Conclusions

In conclusion, our study demonstrates that the gut microbiome in juvenile chum salmon undergoes substantial shifts during early development, driven by the initiation of feeding and seawater transition, with diet and environment exerting significant influence. Our results reveal the potential vulnerability of these early life stages to environmental perturbations, highlighting the importance of considering microbiome dynamics in assessing the impact of climate change on salmonid populations. Future research should focus on elucidating the functional roles of the identified key microbial taxa and extend investigations to other life stages and associated microbial communities. Critically, exploring the potential for microbiome-based interventions, such as targeted probiotic administration, offers a promising way to enhance the resilience of juvenile salmon under rapidly changing environmental conditions.

## Ethics statement

This study was performed according to the Guideline for Care and Use of Animals approved by the Animal Experiment Committee of The University of Tokyo. The present study was carried out in compliance with the Animal Research: Reporting of In Vivo Experiments (ARRIVE) guidelines.

## Use of generative AI in scientific writing

Generative AI (Google Gemini) was used only to check the quality of the writing and improve its readability.

## Funding information

The study was supported by The Ocean Research Promotion Technology Development Program from the Ministry of Education, Culture, Sports, Science, and Technology (MEXT), and the Future Society Initiative Fund from the University of Tokyo (Ocean DNA), Japan.

## CRediT authorship contribution statement

**Subrata Kumar Ghosh:** Conceptualization, Methodology, Investigation, Formal analysis, Visualization, Validation, Writing-original draft. **Marty Kwok-Shing Wong:** Methodology, Technical support, Validation, Writing-review and editing, **Susumu Hyodo:** Methodology, Technical support, Validation, Writing-review and editing, **Koji Hamasaki:** Conceptualization, Methodology, Supervision, Writing-review and editing, Validation, Funding acquisition.

## Declaration of competing interest

The authors declare that they have no known competing financial interests or personal relationships that could have appeared to influence the work reported in this paper.

## Data Availability

The 16S rRNA gene sequencing datasets presented in this study are available in the DDBJ online repositories under the accession number DRA017221.

## Appendix Supplementary materials

Image, application 1

Image, application 2
