# Supplementary material for: Shifting seas and first feeds: gut microbiome dynamics in juvenile chum salmon (Oncorhynchus keta) and their climate vulnerability
Source: Curr Res Microb Sci. 2025 Jul 30;9:100452. doi: 10.1016/j.crmicr.2025.100452 (PMC12355085; doi:10.1016/j.crmicr.2025.100452)
Supplement: Supplementary file 1 [file mmc1.pdf]

Supplementary Data

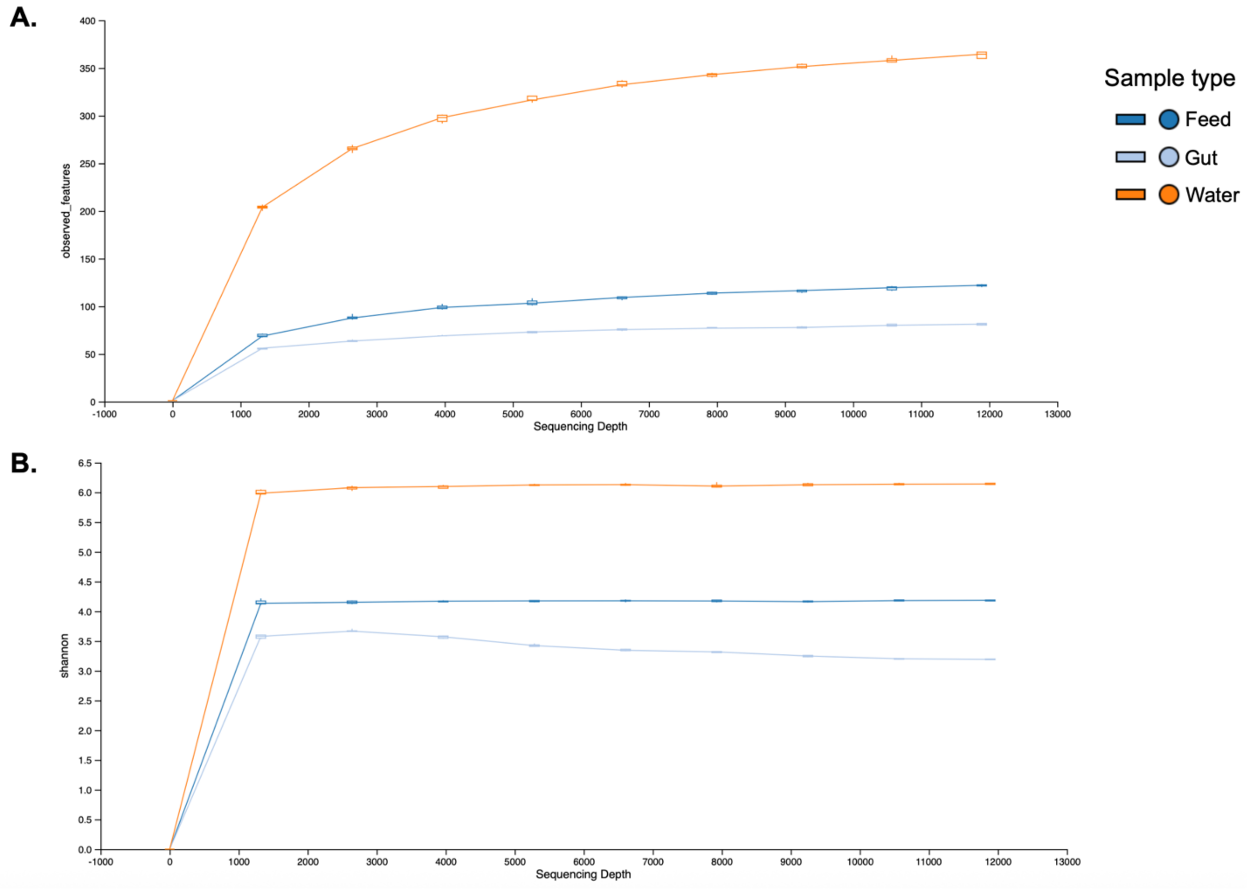

Supplementary Figure (S1): Rarefaction curves for the gut, feed, and water samples. Rarefaction curves were generated for the gut, feed, and water samples based on (A) observed sequence variants (ASVs) and (B) Shannon diversity index measurements for each sample type. The diversity estimation involved rarefying the number of sequences per sample to 11,890 reads. The normalized ASVs were used to plot the rarefaction curves in Qiime2. The colors used to represent the samples are orange for water, dark blue for feed, and sky blue for gut samples.

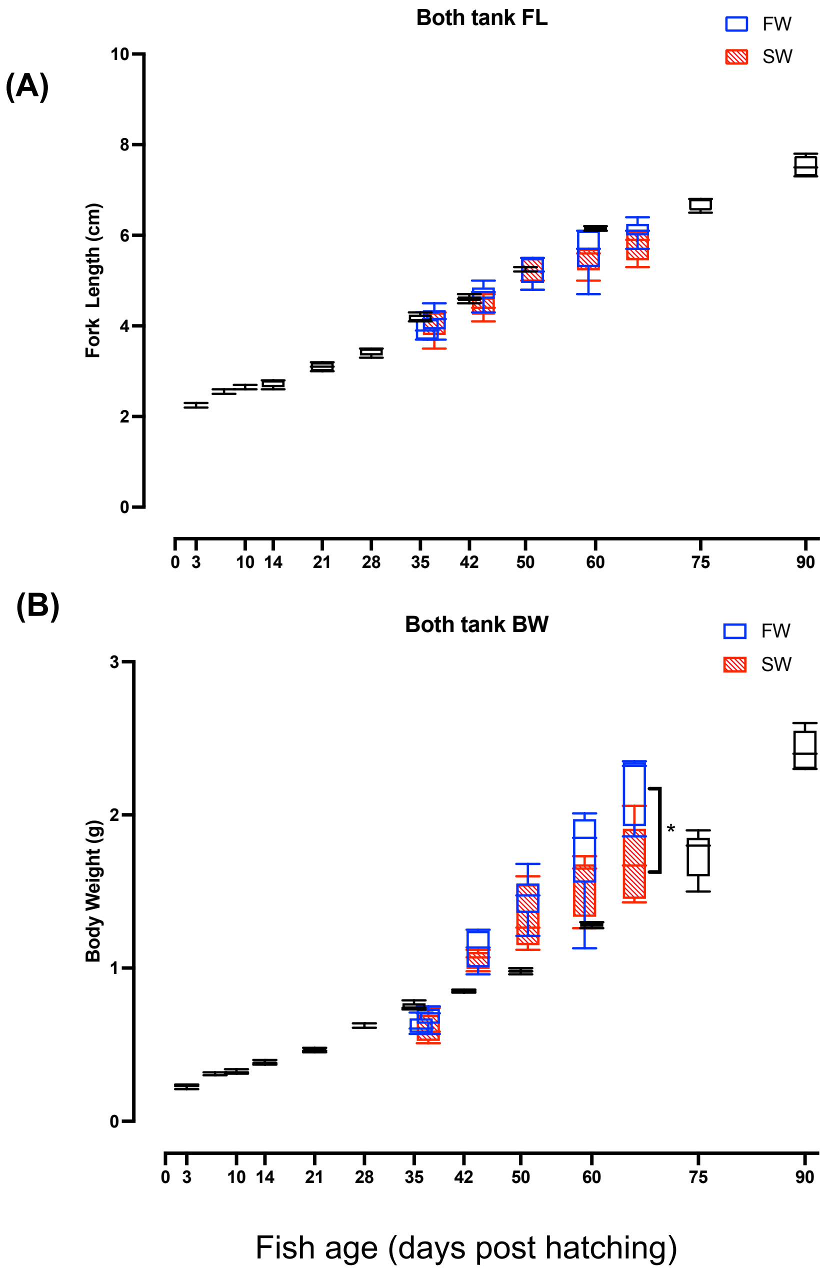

Supplementary Figure (S2): Mean fork lengths (FL) and body weights (BW) of the chum salmon sampled at 3, 7, 10, 14, 21, 28, 35, 36, 37, 42, 44, 50, 51, 59, 60, 66, 75, and 90 days post-hatching (DPH) in freshwater (FW) and seawater (SW) during the study period. Mean fish fork length (cm) and body weight (g) are represented as box plots. At each sampling point, a total of six fish were measured and the average value was considered for generating the plot.

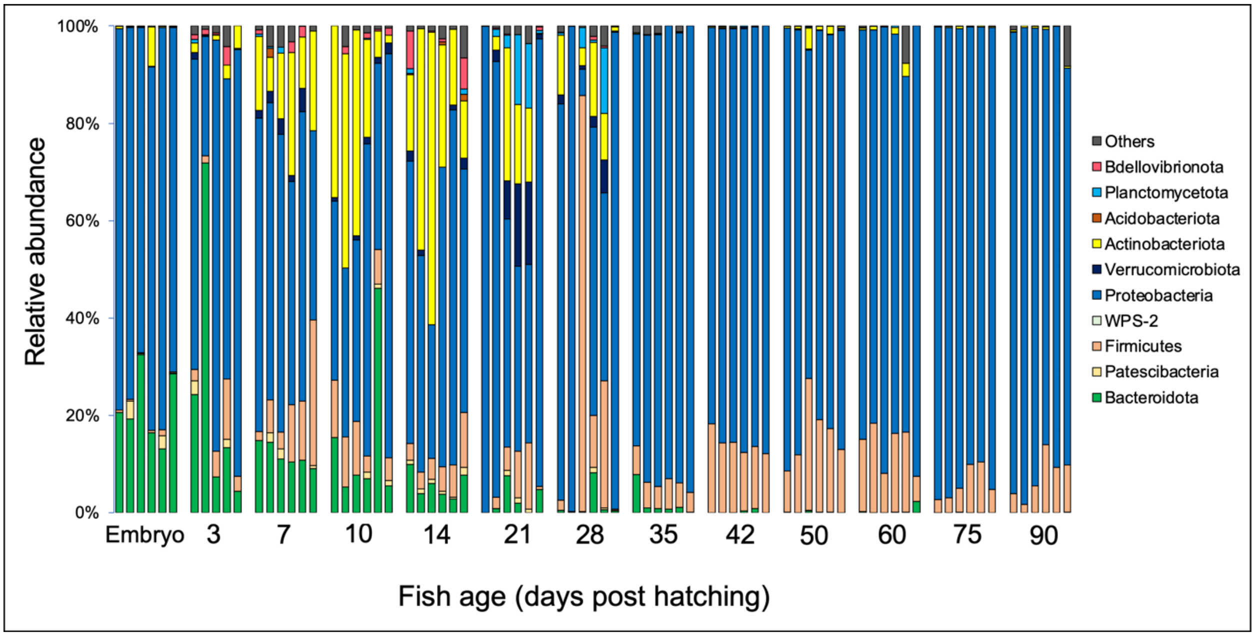

Supplementary Figure (S3): Bacterial community composition of chum salmon at different developmental stages, shown at the phylum level. Bacterial relative abundance data are shown as the value calculated from six replicates of each sample. Phyla that contributed less than 2% were combined and referred to as "others".

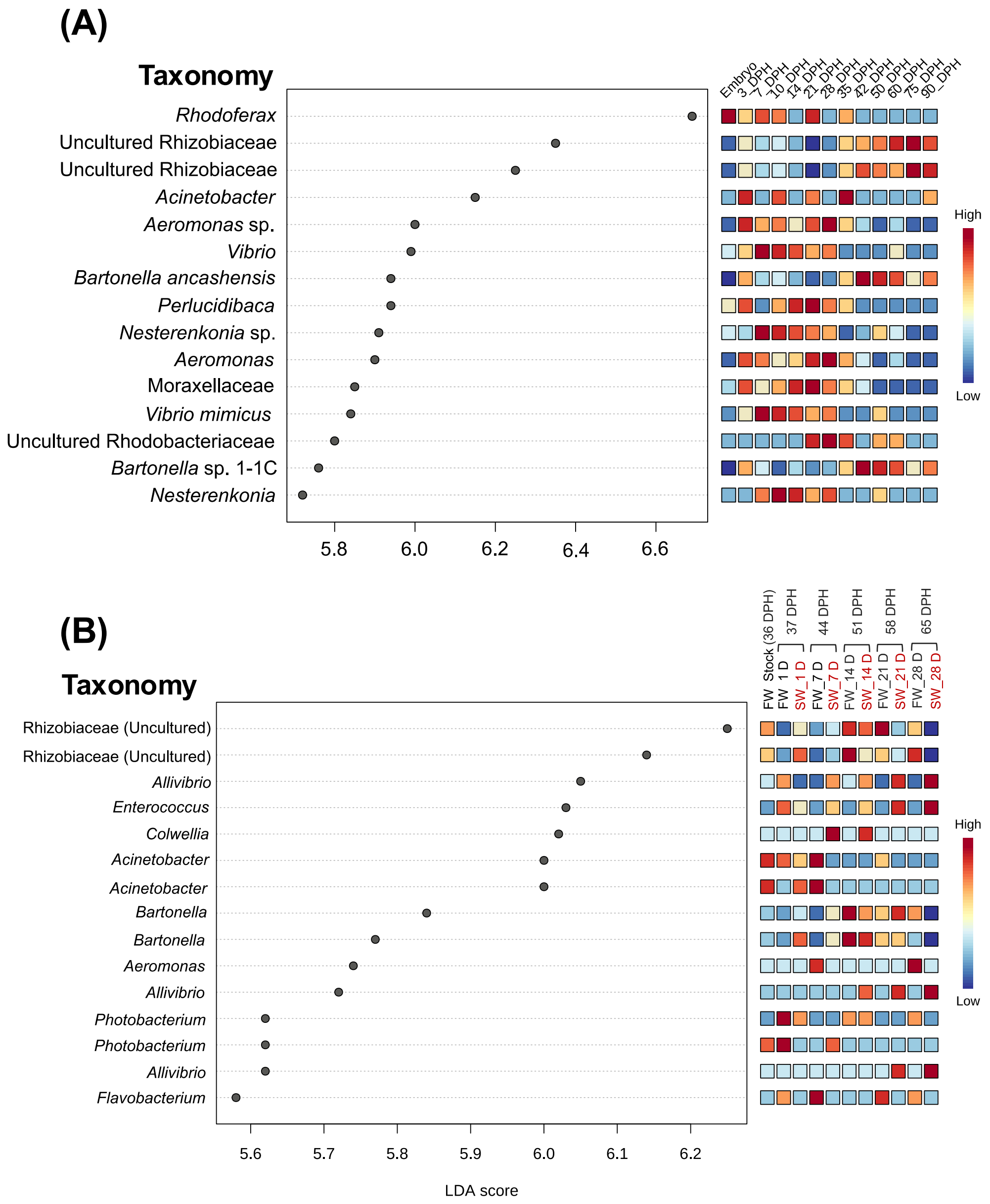

Supplementary Figure (S4): Dot plot showing the significantly abundant taxonomic groups in (A) each of the developmental stages of chum salmon in freshwater, (B) significantly abundant taxonomic groups during different stages of seawater transition, identified based on the Linear discriminant analysis (LDA) effect size (LEfSe). The threshold of the logarithmic LDA score was 3.0, and $p<0.05$ (Kruskal-Wallis test). The developmental stages of fish are denoted as days post-hatching (DPH).

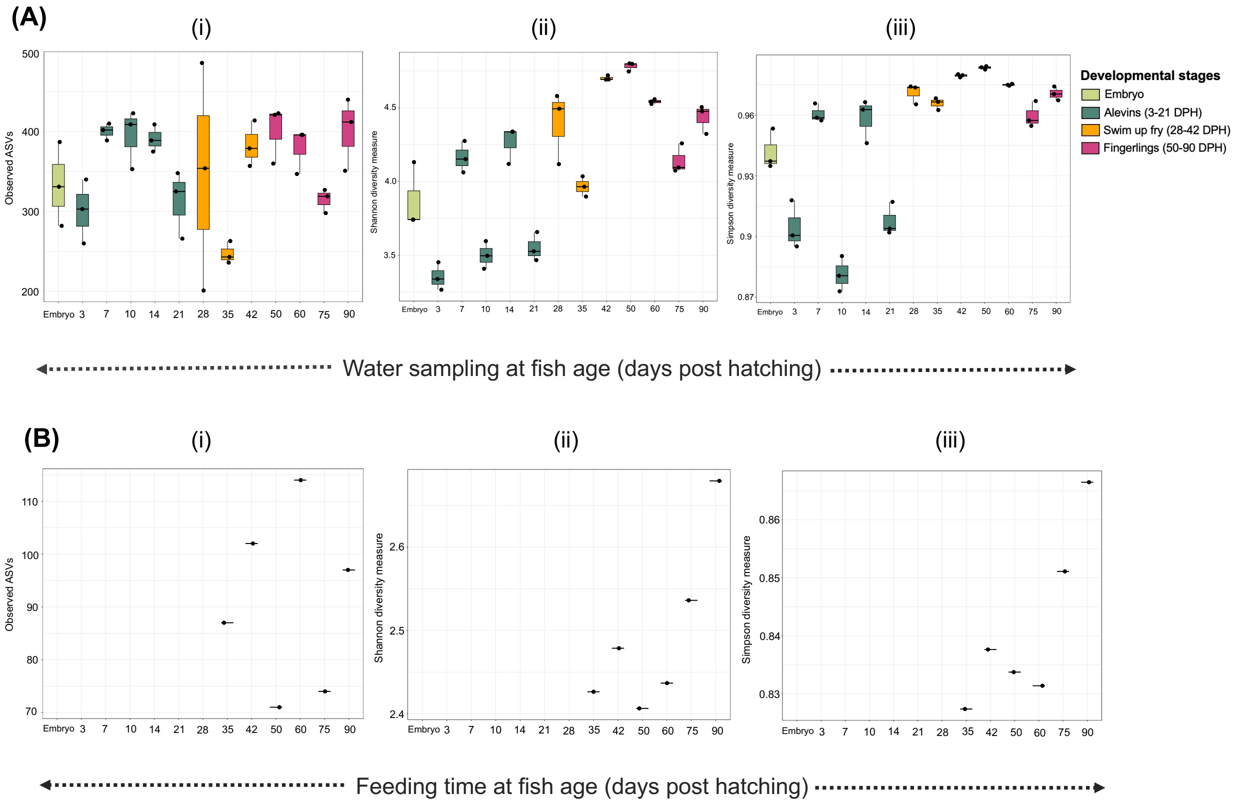

Supplementary Figure (S5): Microbial alpha diversity of environmental samples (A) rearing water and, (B) administered diet at different developmental stages of chum salmon, shown using different alpha diversity indices, (i)=observed ASVs, (ii)=Shannon index, and (iii)=Simpson index. Each color represents a major developmental stage of chum salmon expressed as days post-hatching (DPH). In the estimation of diversity, the number of sequences per sample was rarefied at 11,890 sequence reads, and plots were achieved by processing the ASV table in the R environment (v. 4.2.3, 2022; R Core Team).

(A)

(B)

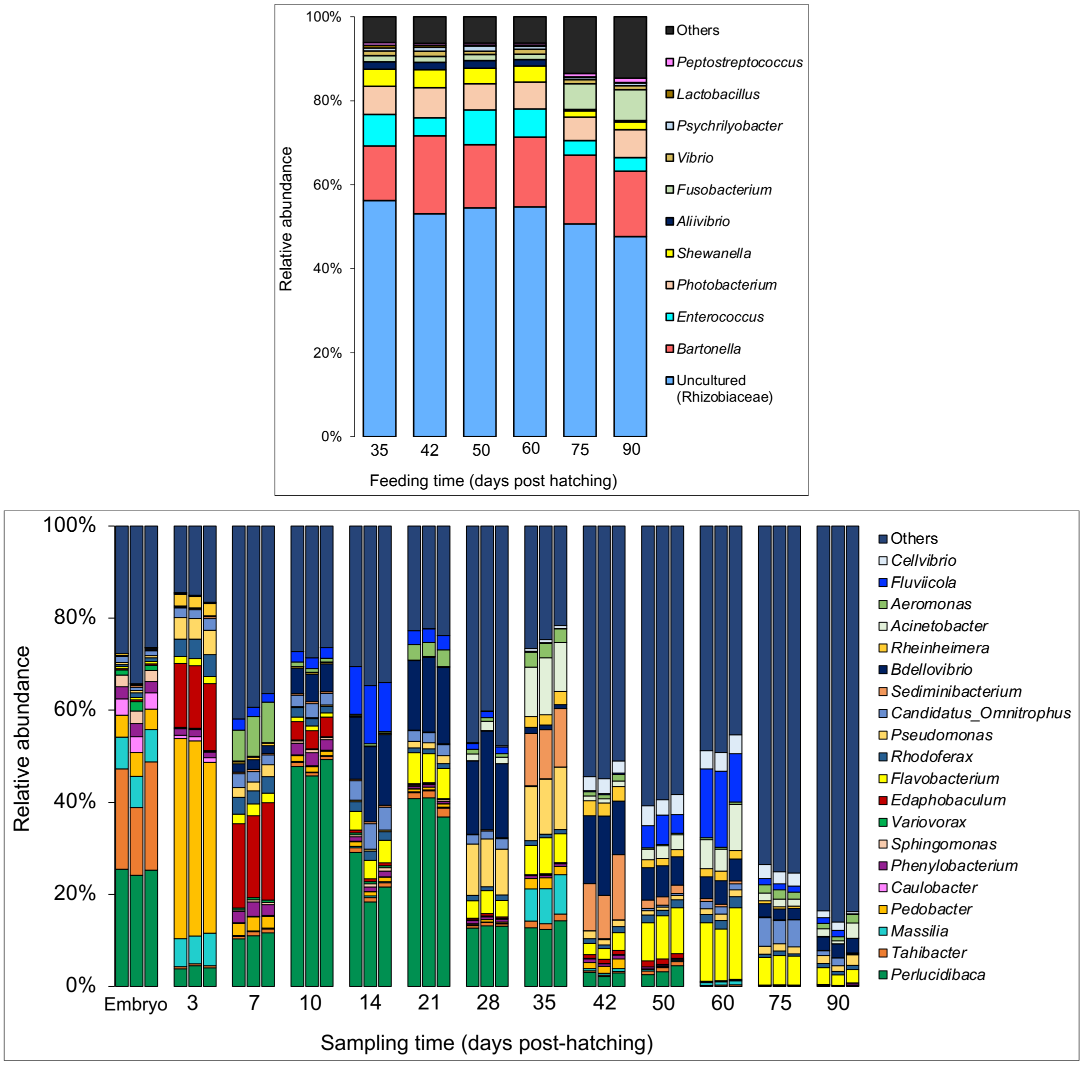

Supplementary Figure (S6): Bacterial community composition of environmental samples at all the sampling points in freshwater: (A) administered diet, and (B) rearing water at different developmental stages of chum salmon, shown at the genus level. Bacterial relative abundance data are shown as the value calculated from three replicates of water and one respective feed sample. Bacterial taxa that are not classified up to the genus level are shown at the highest taxonomic level available.

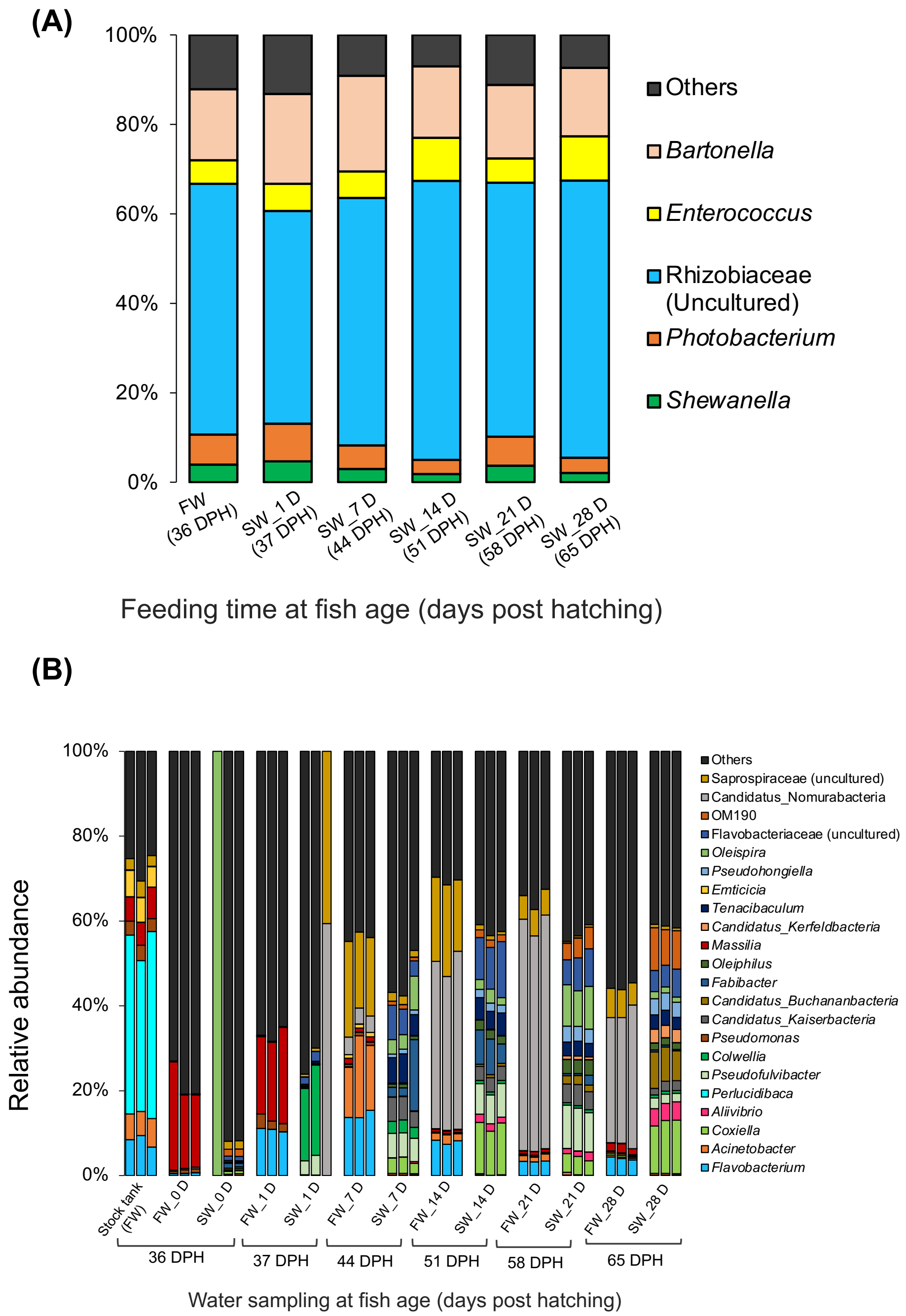

Supplementary Figure (S7): Bacterial community composition of environmental samples at all the sampling points: (A) administered diet, and (B) rearing water at different developmental stages of chum salmon in freshwater and seawater, shown at the genus level. Bacterial relative abundance data are shown as the value calculated from three replicates of water and one respective feed sample. Bacterial taxa that are not classified up to the genus level are shown at the highest taxonomic level available.

Supplementary Table 1: Environmental variables at all the sampling points at different developmental stages of chum salmon in (A)= freshwater (FW) stock tank, and (B)= during the seawater (SW) transition. Temperature, pH, and salinity were measured twice a day in the morning and afternoon, and the values are shown as mean ± standard deviation (SD). Daily water replacement (approximately 30%) with continuous aeration was in place, ensuring a continuous supply of dissolved oxygen (DO); therefore, DO data were not taken daily. The developmental stages of fish are denoted as days post-hatching (DPH).

(A)

Date	Developmental stage	Temperature (°C)	pH	Date	Developmental stage	Temperature (°C)	pH
2022.01.20	Embryo	12 ± 0.05	6.7 ± 0.05	2022.02.03	Sac fry (10 DPH)	12.2 ± 0.1	6.8 ± 0.15
2022.01.21		12	6.8 ± 0.1	2022.02.04		12.2	6.8 ± 0.2
2022.01.22		12.5	6.7 ± 0.4	2022.02.05		12	6.7 ± 0.14
2022.01.23		12	6.8 ± 0.2	2022.02.06		12	6.7 ± 0.1
2022.01.24		12	6.7 ± 0.01				
2022.01.25		12	6.8 ± 0.04				
2022.01.26	Sac fry (3 DPH)	12 ± 0.1	6.6 ± 0.14	2022.02.07	Sac fry (14 DPH)	12	6.8 ± 0.15
2022.01.27		12	6.8 ± 0.2	2022.02.08		12	6.7 ± 0.03
2022.01.28		12	6.6 ± 0.1	2022.02.09		12.2	6.8 ± 0.02
2022.01.29		12.2	6.9 ± 0.1	2022.02.10		12.2	6.8 ± 0.05
2022.01.30		12	6.8 ± 0.4	2022.02.11		12.2	6.8 ± 0.03
				2022.02.12		12.2	6.8 ± 0.02
				2022.02.13		12.2	6.7 ± 0.15
				2022.02.14		12.2	6.7 ± 0.03
2022.01.31	Sac fry (7 DPH)	12.5 ± 0.1	6.7 ± 0.11	2022.02.15	Swim-up fry (21 DPH)	12.2 ± 0.05	6.8 ± 0.11
2022.02.01		12.2	6.7 ± 0.03	2022.02.16		12.2	6.7 ± 0.03
2022.02.02		12.2	6.8 ± 0.02	2022.02.17		12.2	6.8 ± 0.05
				2022.02.18		12.2	6.8 ± 0.03
				2022.02.19		12.2	6.7 ± 0.03
				2022.02.20		12.0	6.7 ± 0.05
				2022.02.21		12.2	6.7 ± 0.05
2022.02.22	Swim-up fry 28 DPH	12.1 ± 0.1	6.8 ± 0.03	2022.03.25	Fingerlings (60 DPH)	12.2	6.9 ± 0.1
2022.02.23		12.2	6.7 ± 0.3	2022.03.26		12	6.8 ± 0.03

2022.02.24		12.2	6.7 ± 0.03	2022.03.27		12	6.8 ± 0.02
2022.02.25		12.2	6.8 ± 0.02	2022.03.28		12	6.8 ± 0.03
2022.02.26		12.2	6.7 ± 0.07	2022.03.29		12.2	6.7 ± 0.03
2022.02.27		12	6.7 ± 0.05	2022.03.30		12.2	6.7 ± 0.05
2022.02.28		12.2	6.7 ± 0.03	2022.03.31		12.2	6.7 ± 0.05
				2022.04.01		12.2	6.8 ± 0.03
				2022.04.02		12.2	6.7 ± 0.03
				2022.04.03		12.2	6.7 ± 0.01
				2022.04.04		12.2	6.7 ± 0.01
				2022.04.05		12.2	6.7 ± 0.04
				2022.04.06		12	6.7 ± 0.03
				2022.04.07		12.2	6.7 ± 0.05
2022.03.01	Swim-up fry (35 DPH)	12.2	6.8 ± 0.07	2022.04.08	Fingerlings (75 DPH)	12.2 ± 0.05	6.8 ± 0.02
2022.03.02		12.2	6.8 ± 0.05	2022.04.09		12.2	6.8 ± 0.03
2022.03.03		12.2	6.8 ± 0.03	2022.04.10		12.2	6.7 ± 0.03
2022.03.04		12.2	6.8 ± 0.02	2022.04.11		12.2	6.7 ± 0.03
2022.03.05		12.2	6.7 ± 0.07	2022.04.12		12.2	6.7 ± 0.05
2022.03.06		12.2	6.7 ± 0.03	2022.04.13		12.2	6.7 ± 0.01
2022.03.07		12.2	6.7 ± 0.05	2022.04.14		12	6.7 ± 0.03
				2022.04.15		12	6.7 ± 0.03
				2022.04.16		12.2	6.7 ± 0.03
				2022.04.17		12.2	6.7 ± 0.05
				2022.04.18		12.2	6.7 ± 0.01
				2022.04.19		12.2	6.7 ± 0.01
				2022.04.20		12.2	6.7 ± 0.03
				2022.04.21		12.2	6.7 ± 0.03
2022.03.08	Fingerlings (42 DPH)	12.2	6.8 ± 0.06	2020.4.22	Fingerlings (90 DPH)	12.2 ± 0.2	6.8 ± 0.15
2022.03.09		12.2	6.7 ± 0.09				
2022.03.10		12	6.8 ± 0.02				
2022.03.11		12.2	6.7 ± 0.05				
2022.03.12		12.2	6.7 ± 0.05				
2022.03.13		12.2	6.7 ± 0.03				
2022.03.14		12.2	6.7 ± 0.03				
2022.03.15		12	6.7 ± 0.03				
2022.03.16	Fingerlings (50 DPH)	12 ± 0.11	6.8 ± 0.02				
2022.03.17		12.2	6.8 ± 0.04				
2022.03.18		12.2	6.8 ± 0.02				
2022.03.19		12.2	6.7 ± 0.1				

2022.03.20	12.2	6.7 ± 0.1
2022.03.21	12.2	6.7 ± 0.05
	12.2	6.7 ± 0.03
2022.03.22	12.2	6.7 ± 0.03
2022.03.23	12.2	6.7 ± 0.01
2022.03.24		

(B)

Date	Developmental stage (DPH)	Temperature (°C)		pH	Salinity (PSU)
2022.03.02	36 DPH	12.2 (FW stock tank)		6.9 ± 0.05	-
		**FW transfer tank**	**SW transfer tank**		**SW transfer tank**
2022.03.03	37 DPH (FW.1D & SW.1D)	12 ± 0.05	12.1 ± 0.1	6.7 ± 0.05	34.3 ± 0.5
2022.03.04		12	12.2	6.8 ± 0.1	35
2022.03.05		12.5	12.2	6.7 ± 0.4	34.3 ± 0.5
2022.03.06		12	12.2	6.8 ± 0.2	34.6 ± 0.5
2022.03.07		12	12.2	6.7 ± 0.01	34.6 ± 0.5
2022.03.08		12	12	6.8 ± 0.04	34.3 ± 0.5
2022.03.09		12.2	12.2	6.7 ± 0.4	35
2022.03.10	44 DPH (FW.7D & SW.7D)	12 ± 0.1	12.2	6.6 ± 0.14	34.3 ± 0.5
2022.03.11		12	12	6.8 ± 0.2	34.6 ± 0.5
2022.03.12		12	12.2	6.6 ± 0.1	34.6 ± 0.5
2022.03.13		12.2	12.2	6.9 ± 0.1	34.3 ± 0.5
2022.03.14		12	12.2	6.8 ± 0.4	34.6 ± 0.5
2022.03.15		12.2	12	6.8 ± 0.2	34.3 ± 0.5
2022.03.16		12.2	12.2	6.6 ± 0.1	34.6 ± 0.5
2022.03.17	51 DPH (FW.14D & SW.14D)	12.5 ± 0.1	12.2	6.7 ± 0.11	34.6 ± 0.5
2022.03.18		12.2	12.2	6.7 ± 0.01	35
2022.03.19		12.2	12.2	6.8 ± 0.04	34.8 ± 0.5
2022.03.20		12	12.2	6.8 ± 0.2	34.8 ± 0.5
2022.03.21		12.2	12.1	6.7 ± 0.01	34.6 ± 0.5
2022.03.22		12	12.2	6.8 ± 0.04	34.8 ± 0.5
2022.03.23		12	12.2	6.7 ± 0.4	35

2022.03.24<br>2022.03.25<br>2022.03.26<br>2022.03.27<br>2022.03.28<br>2022.03.29<br>2022.03.30	59 DPH<br>(FW.21D & SW.21D)	12.1 ± 0.1<br>12.2<br>12.2<br>12.2<br>12.2<br>12<br>12.2	12.2<br>12.2<br>12.2<br>12.2<br>12.1<br>12.2<br>12.2	6.8 ± 0.1<br>6.8 ± 0.04<br>6.8 ± 0.2<br>6.9 ± 0.1<br>6.8 ± 0.4<br>6.7 ± 0.01<br>6.8 ± 0.04	34.6 ± 0.57<br>34.6 ± 0.57<br>34.3 ± 0.57<br>34.6 ± 0.5<br>34.6 ± 0.5<br>34.3 ± 0.5<br>34.7 ± 0.5
2022.03.31	66 DPH<br>(FW.28 D & SW.28 D)	12.2		6.8 ± 0.12	35

Supplementary Table 2: (A) Test results of statistical analyses of bacterial alpha diversity indices (Shannon diversity index and observed ASVs) across samples using a fixed factor of fish age (days post hatch; DPH) are shown by one-way ANOVA. (B) Test results of non-parametric permutation-based multivariate statistical analysis of beta diversity of the intestinal microbiota of chum salmon across age (days post hatch; DPH), and fish type (freshwater/seawater) based on Bray-Curtis distance. (C) Pairwise test results of non-parametric permutation-based multivariate statistical analysis of beta diversity of the intestinal microbiota of chum salmon across age (days post hatch; DPH) based on Bray-Curtis distance. Test statistics were calculated using up to 9,999 permutations; the significant groups are listed here. *-Indicates a rejection of the null hypothesis of no differences among groups ($p < 0.05$ (*), $p < 0.01$ (**), $p < 0.001$ (***)).

(A)

**One-way ANOVA**													
	Shannon diversity index						Observed ASVs						
Factor	*df*	Sum sq.	Mean Sq.	F value	Pr(>F)	Signif.	Factor	*df*	Sum sq.	Mean Sq.	F value	Pr(>F)	Signif.
Fish age	12	39.61	3.301	6.129	0.000000552	***	Fish age	31	105055	3389	15.17	0.0002	***
Residuals	63	33.93	0.539				Residuals	90	20111	223			
Signif. Codes: '***' 0.001 '**' 0.01 '*' 0.05 '.' 0.1 ' ' 1													

(B)

Statistical test & factor	*df*	Sum sq.	Mean Sq.	F.Model	R^2	Pr(>F)	Significance
PERMANOVA (Host age)	2	1.477302792	0.738651396	56.89017356	0.674199719	0.00019996	***
PERMANOVA (Fish type)	2	0.428247925	0.214123963	16.49160817	0.195440388	0.00019996	***
Signif. Codes: '***' 0.001 '**' 0.01 '*' 0.05 '.' 0.1 ' ' 1							

(C)

Groups compared	R^2	P value	Groups compared	R^2	P value
Embryo vs 14 DPH	0.140193	*0.024	10 DPH vs 28 DPH	0.214456	*0.01
Embryo vs 35 DPH	0.197304	*0.016	10 DPH vs 35 DPH	0.276847	**0.005
Embryo vs 42 DPH	0.208858	*0.022	10 DPH vs 42 DPH	0.291398	**0.003
Embryo vs 42 DPH	0.208858	*0.014	10 DPH vs 50 DPH	0.366252	**0.002
Embryo vs 50 DPH	0.281283	**0.008	10 DPH vs 60 DPH	0.33595	**0.006
Embryo vs 60 DPH	0.255894	**0.009	10 DPH vs 75 DPH	0.385723	***0.001
Embryo vs 75 DPH	0.292875	**0.004	10 DPH vs 90 DPH	0.286957	**0.002
Embryo vs 90 DPH	0.198634	*0.016	14 DPH vs 35 DPH	0.25444	**0.003
3 DPH vs 14 DPH	0.185534	**0.009	14 DPH vs 42 DPH	0.271927	***0.003
3 DPH vs 21 DPH	0.169640	**0.006	14 DPH vs 50 DPH	0.331551	**0.005
3 DPH vs 28 DPH	0.258240	**0.007	14 DPH vs 60 DPH	0.30351	**0.003
3 DPH vs 35 DPH	0.327989	**0.008	14 DPH vs 75 DPH	0.349096	***0.001
3 DPH vs 42 DPH	0.336800	***0.001	14 DPH vs 90 DPH	0.261655	**0.002
3 DPH vs 50 DPH	0.419787	**0.005	21 DPH vs 35 DPH	0.196503	**0.009
3 DPH vs 60 DPH	0.377679	**0.007	21 DPH vs 42 DPH	0.224041	**0.003
3 DPH vs 75 DPH	0.433252	**0.005	21 DPH vs 50 DPH	0.26911	*0.014
3 DPH vs 90 DPH	0.328829	**0.002	21 DPH vs 60 DPH	0.254236	**0.007
7 DPH vs 35 DPH	0.229040	**0.006	21 DPH vs 75 DPH	0.294639	**0.003
7 DPH vs 42 DPH	0.243957	**0.005	21 DPH vs 90 DPH	0.211906	**0.005
7 DPH vs 50 DPH	0.309704	*0.015	28 DPH vs 50 DPH	0.243428	*0.015
7 DPH vs 60 DPH	0.286048	**0.006	28 DPH vs 60 DPH	0.190484	*0.017
7 DPH vs 75 DPH	0.330270	***0.001	28 DPH vs 75 DPH	0.225893	**0.004
7 DPH vs 90 DPH	0.238823	**0.004			

Supplementary Table 3: One-way Similarity Percentage Analysis (SIMPER) of the most discriminatory bacterial taxa detected in the intestine of chum salmon at various ages (days post hatch; DPH). Before analysis, ASVs were filtered to remove those detected in less than three individuals. Only those ASVs that accounted for the top 90% of between-group dissimilarity are listed.

**Group1 Group 2 (Dissimilarity)**	**Discriminatory Bacterial Taxa (Order ∣ Genus)**	**Relative abundance (%)**		**Dissimilarity Contribution (%)**
		**Group 1 Mean**	**Group 2 Mean**	
Embryo 3 DPH<br>**(95.06%)**	Burkholderiales ∣ *Rhodoferax*<br>Burkholderiales ∣ *Undibacterium*	10.32<br>2.5	0.24<br>0.27	8.82<br>2
Embryo 7 DPH<br>**(96.72%)**	Burkholderiales ∣ *Rhodoferax*<br>Vibrionales ∣ *Vibrio*<br>Micrococcales ∣ *Nesterenkonia*	10.32<br>0.03<br>0.08	0.42<br>2.82<br>2.52	8.47<br>2.43<br>2.12
Embryo 10 DPH<br>**(96.84%)**	Burkholderiales ∣ *Rhodoferax*<br>Burkholderiales ∣ *Undibacterium*<br>Micrococcales ∣ *Nesterenkonia*	10.32<br>2.5<br>0.08	0.12<br>0<br>2.21	8.97<br>2.19<br>1.92
Embryo 14 DPH<br>**(97.02%)**	Burkholderiales ∣ *Rhodoferax*<br>Burkholderiales ∣ *Undibacterium*<br>Micrococcales ∣ *Nesterenkonia*	10.32<br>2.5<br>0.58	0.16<br>0<br>2.4	8.83<br>2.18<br>2.06
Embryo 21 DPH<br>**(95.23%)**	Burkholderiales ∣ *Rhodoferax*<br>Aeromonadales ∣ *Aeromonas*<br>Pseudomonadales ∣ *Perlucidibaca*<br>Burkholderiales ∣ *Undibacterium*	10.32<br>0.03<br>0.08<br>4.66	1.06<br>1.98<br>2.38<br>0.19	9.53<br>2.62<br>2.53<br>4.51
Embryo 28 DPH<br>**(97.61%)**	Burkholderiales ∣ *Rhodoferax*<br>Aeromonadales ∣ *Aeromonas*<br>Burkholderiales ∣ *Undibacterium*	10.32<br>0.03<br>4.66	0.16<br>5.12<br>0	10.35<br>6.59<br>4.73
Embryo 35 DPH<br>**(95.94%)**	Burkholderiales ∣ *Rhodoferax*<br>Pseudomonadales ∣ *Acinetobacter*<br>Rhizobiales ∣ Rhizobiaceae*<br>Burkholderiales ∣ *Undibacterium*<br>Rhizobiales ∣ *Bartonella*	10.32<br>0.07<br>0.77<br>2.5<br>0.19	0.13<br>8.24<br>7.56<br>0<br>2.52	10.41<br>8.45<br>7.03<br>2.55<br>2.37
Embryo 42 DPH<br>**(96.54%)**	Burkholderiales ∣ *Rhodoferax*<br>Rhizobiales ∣ Rhizobiaceae*<br>Rhizobiales ∣ *Bartonella*<br>Burkholderiales ∣ *Undibacterium*<br>Lactobacillales ∣ *Enterococcus*	10.32<br>0.77<br>0.34<br>2.5<br>0.08	0<br>10.33<br>7.66<br>0<br>2.48	12.84<br>11.95<br>9.05<br>3.11<br>3.01
Embryo 50 DPH<br>**(96.47%)**	Burkholderiales ∣ *Rhodoferax*<br>Rhizobiales ∣ Rhizobiaceae*<br>Rhizobiales ∣ *Bartonella*	10.32<br>0.77<br>0.34	0.02<br>10.69<br>6.79	11.87<br>11.52<br>7.49
Embryo 60 DPH<br>**(96.47%)**	Burkholderiales ∣ *Rhodoferax*<br>Rhizobiales ∣ Rhizobiaceae*<br>Rhizobiales ∣ *Bartonella*<br>Burkholderiales ∣ *Undibacterium*	10.32<br>0.77<br>0.19<br>2.5	0<br>10.21<br>3.15<br>0	11.94<br>11.06<br>3.42<br>2.89
Embryo 75 DPH<br>**(96.5%)**	Burkholderiales ∣ *Rhodoferax*<br>Rhizobiales ∣ Rhizobiaceae*<br>Rhizobiales ∣ *Bartonella*	10.32<br>0.77<br>0.19	0<br>12.41<br>2.9	13.41<br>15.14<br>3.52

Embryo 90 DPH **(96.89%)**	Rhizobiales│Rhizobiaceae*<br>Burkholderiales│*Rhodoferax*<br>Rhizobiales│*Bartonella*	0.77<br>10.32<br>0.34	11.6<br>0<br>5.89	13.3<br>12.46<br>6.59
3 DPH 7 DPH **(90.23%)**	Micrococcales│*Nesterenkonia*<br>Vibrionales│*Vibrio*<br>Sphingobacteriales│*Pedobacter*	0.12<br>0.61<br>1.75	2.52<br>5.18<br>0.15	1.93<br>3.68<br>1.21
3 DPH 10 DPH **(90.13%)**	Micrococcales│*Nesterenkonia*<br>Vibrionales│*Vibrio*<br>Alteromonadales│*Colwellia*	0.12<br>0.06<br>1.49	4.15<br>1.88<br>0.72	3.35<br>1.48<br>1.28
3 DPH 21 DPH **(92.89%)**	Pseudomonadales│*Perlucidibaca*<br>Aeromonadales│*Aeromonas*	1.58<br>1.51	4.35<br>1.98	4.24<br>2.05
3 DPH 28 DPH **(92.65%)**	Aeromonadales│*Aeromonas*<br>Rhizobiales│Rhizobiaceae*	2.68<br>2.23	5.12<br>0.45	5.56<br>1.64
7 DPH 21 DPH **(91.96%)**	Pseudomonadales│*Perlucidibaca*<br>Aeromonadales│*Aeromonas*<br>Vibrionales│*Vibrio*	0<br>0.55<br>2.82	2.38<br>1.98<br>0.71	2.17<br>2.05<br>2.01
7 DPH 28 DPH **(91.69%)**	Aeromonadales│*Aeromonas*<br>Vibrionales│*Vibrio*	1.24<br>2.82	5.12<br>0.76	5.49<br>2.02
7 DPH 35 DPH **(91.48%)**	Pseudomonadales│*Acinetobacter*<br>Vibrionales│*Vibrio*<br>Rhizobiales│Rhizobiaceae*<br>Micrococcales│*Nesterenkonia*	0<br>2.82<br>1.38<br>2.52	8.24<br>0<br>4.02<br>0.04	7.45<br>2.56<br>2.42<br>2.25
7 DPH 42 DPH **(91.7%)**	Rhizobiales│Rhizobiaceae*<br>Rhizobiales│*Bartonella*<br>Vibrionales│*Vibrio*	2.48<br>0.54<br>2.82	10.33<br>4.11<br>0.03	8.42<br>3.79<br>3.01
7 DPH 50 DPH **(91.12%)**	Rhizobiales│Rhizobiaceae*<br>Rhizobiales│*Bartonella*<br>Vibrionales│*Vibrio*	2.48<br>0.54<br>2.82	10.69<br>3.65<br>0.11	8.32<br>3.16<br>2.77
7 DPH 60 DPH **(91.99%)**	Rhizobiales│Rhizobiaceae*<br>Vibrionales│*Vibrio*<br>Rhizobiales│*Bartonella*	2.48<br>5.18<br>0.54	10.21<br>0.27<br>3.15	8.17<br>4.97<br>2.62
7 DPH 75 DPH **(92.20%)**	Rhizobiales│Rhizobiaceae*<br>Vibrionales│*Vibrio*<br>Micrococcales│*Nesterenkonia*	2.48<br>5.18<br>2.52	12.41<br>0<br>0.06	10.91<br>5.76<br>2.72
7 DPH 90 DPH **(92.60%)**	Rhizobiales│Rhizobiaceae*<br>Vibrionales│*Vibrio*<br>Rhizobiales│*Bartonella*	2.48<br>2.82<br>0.54	11.6<br>0.04<br>3.14	9.57<br>2.9<br>2.65
10 DPH 28 DPH **(90.45%)**	Aeromonadales│*Aeromonas*<br>Micrococcales│*Nesterenkonia*<br>Vibrionales│*Vibrio*	0.48<br>2.21<br>1.97	5.12<br>0.78<br>0.76	5.78<br>1.7<br>1.47
10 DPH 35 DPH **(91.48%)**	Pseudomonadales│*Acinetobacter*<br>Rhizobiales│Rhizobiaceae*<br>Micrococcales│*Nesterenkonia*	0.25<br>1.93<br>2.21	8.24<br>7.56<br>0.04	7.52<br>5.35<br>2.04
10 DPH 42 DPH **(92.53%)**	Rhizobiales│Rhizobiaceae*<br>Rhizobiales│*Bartonella*<br>Lactobacillales│*Enterococcus*<br>Micrococcales│*Nesterenkonia*	1.93<br>1.02<br>0.24<br>2.21	10.33<br>7.66<br>2.48<br>0.14	9.25<br>7.13<br>2.4<br>2.34
10 DPH 50 DPH	Rhizobiales│Rhizobiaceae*	1.93	10.69	9.13

**(91.46%)**	Rhizobiales∣*Bartonella*<br>Lactobacillales∣*Enterococcus*	1.02<br>0.34	6.79<br>4.96	5.94<br>4.63
10 DPH 60 DPH<br>**(91.84%)**	Rhizobiales∣Rhizobiaceae*<br>Rhizobiales∣*Bartonella*<br>Micrococcales∣*Nesterenkonia*	1.93<br>1.02<br>2.21	10.21<br>5.53<br>0.32	8.95<br>4.52<br>2.09
10 DPH 75 DPH<br>**(92.94%)**	Rhizobiales∣Rhizobiaceae*<br>Rhizobiales∣*Bartonella*<br>Micrococcales∣*Nesterenkonia*	1.93<br>1.02<br>2.21	12.41<br>5.51<br>0.06	11.84<br>5.01<br>2.47
10 DPH 90 DPH<br>**(93.64%)**	Rhizobiales∣Rhizobiaceae*<br>Rhizobiales∣*Bartonella*	1.93<br>3.23	11.6<br>5.89	10.36<br>7.34
14 DPH 90 DPH<br>**(90.41%)**	Rhizobiales∣Rhizobiaceae*<br>Rhizobiales∣*Bartonella*<br>Micrococcales∣*Nesterenkonia*	2.51<br>1.09<br>2.4	11.6<br>5.89<br>0	9.97<br>5.08<br>2.63
21 DPH 35 DPH<br>**(92.97%)**	Pseudomonadales∣*Acinetobacter*<br>Rhizobiales∣Rhizobiaceae*<br>Pseudomonadales∣*Perlucidibaca*<br>Aeromonadales∣*Aeromonas*	0.14<br>0.54<br>2.38<br>1.98	8.24<br>7.56<br>0.9<br>0.6	8.34<br>7.33<br>2.62<br>2.49
21 DPH 42 DPH<br>**(95.84%)**	Rhizobiales∣Rhizobiaceae*<br>Rhizobiales∣*Bartonella*<br>Aeromonadales∣*Aeromonas*<br>Lactobacillales∣*Enterococcus*<br>Pseudomonadales∣*Perlucidibaca*	0.54<br>0.38<br>3.55<br>0<br>2.38	10.33<br>7.66<br>0.39<br>2.48<br>0	12.2<br>8.99<br>6.13<br>3.06<br>3.05
21 DPH 50 DPH<br>**(95.21%)**	Rhizobiales∣Rhizobiaceae*<br>Rhizobiales∣*Bartonella*<br>Aeromonadales∣*Aeromonas*<br>Lactobacillales∣*Enterococcus*	0.54<br>0.38<br>1.98<br>0.08	10.69<br>6.79<br>0.02<br>2.67	11.7<br>7.44<br>3.11<br>2.9
21 DPH 60 DPH<br>**(96.18%)**	Rhizobiales∣Rhizobiaceae*<br>Rhizobiales∣*Bartonella*<br>Aeromonadales∣*Aeromonas*	0.54<br>0.24<br>1.98	10.21<br>3.15<br>0.17	11.15<br>3.37<br>3.08
21 DPH 75 DPH<br>**(97.47%)**	Rhizobiales∣Rhizobiaceae*<br>Aeromonadales∣*Aeromonas*<br>Rhizobiales∣*Bartonella*	0.54<br>1.98<br>0.38	12.41<br>0<br>5.51	15.15<br>3.65<br>6.63
21 DPH 90 DPH<br>**(97.31%)**	Rhizobiales∣Rhizobiaceae*<br>Rhizobiales∣*Bartonella*<br>Aeromonadales∣*Aeromonas*	0.54<br>0.38<br>1.98	11.6<br>5.89<br>0.02	13.36<br>6.49<br>3.31
28 DPH 35 DPH<br>**(92.7%)**	Pseudomonadales∣*Acinetobacter*<br>Rhizobiales∣Rhizobiaceae*<br>Aeromonadales∣*Aeromonas*<br>Rhizobiales∣*Bartonella*	0<br>1.01<br>5.12<br>0.52	8.24<br>7.56<br>1.45<br>4.76	8.85<br>7.29<br>6.65<br>4.62
28 DPH 42 DPH<br>**(94.8%)**	Rhizobiales∣Rhizobiaceae*<br>Rhizobiales∣*Bartonella*<br>Aeromonadales∣*Aeromonas*<br>Lactobacillales∣*Enterococcus*	1.01<br>0.52<br>5.12<br>0.25	10.33<br>7.66<br>0.39<br>4.66	12.5<br>9.39<br>8.76<br>5.78
28 DPH 50 DPH<br>**(94.14%)**	Rhizobiales∣Rhizobiaceae*<br>Rhizobiales∣*Bartonella*<br>Aeromonadales∣*Aeromonas*<br>Rhizobiales∣*Bartonella*	1.01<br>0.24<br>5.12<br>0.32	10.69<br>3.65<br>0.02<br>5.81	11.98<br>4.22<br>8.02<br>6.62
28 DPH 60 DPH<br>**(94.87%)**	Rhizobiales∣Rhizobiaceae*<br>Aeromonadales∣*Aeromonas*	1.01<br>5.12<br>0.24	10.21<br>0.31<br>3.15	11.57<br>8.06<br>3.59

	Rhizobiales│*Bartonella*			
28 DPH 75 DPH<br>**(95.99%)**	Rhizobiales│Rhizobiaceae*<br>Aeromonadales│*Aeromonas*<br>Rhizobiales│*Bartonella*	1.01<br>5.12<br>0.52	12.41<br>0.04<br>5.51	15.71<br>9.24<br>6.93
28 DPH 90 DPH<br>**(96.06%)**	Rhizobiales│Rhizobiaceae*<br>Aeromonadales│*Aeromonas*<br>Rhizobiales│*Bartonella*	1.01<br>5.12<br>0.52	11.6<br>0.08<br>5.89	13.77<br>8.44<br>6.75

Supplementary Table 4: Statistics of Illumina MiSeq sequencing data demonstrate the diverse sample types (gut, feed, water), their replications, the corresponding sequences that remain after rarefaction and quality filtering, and the number of ASVs per sample at various stages in freshwater (FW) and seawater (SW). The developmental stages of fish are indicated by days post-hatching (DPH), where D represents a day.

Sample type	Repliaction	Filtered sequences	No. of ASVs	Sample type	Repliaction	Filtered sequences	No. of ASVs	Sample type	Repliaction	Filtered sequences	No. of ASVs
Embryo (FW)	R-1	1,04,811	64	Gut (14 DPH)	R-1	18,407	132	Gut (35 DPH)	R-6	60,215	73
	R-2	1,18,356	45		R-2	14,846	51	Gut (42 DPH)	R-1	13,304	44
	R-3	1,35,693	70		R-3	13,957	54		R-2	95,262	46
	R-4	82,881	65		R-4	15,592	57		R-3	83,073	34
	R-5	86,364	56		R-5	15,074	62		R-4	88,039	46
	R-6	78,870	57		R-6	18,993	108		R-5	12,283	39
Gut (3 DPH)	R-1	17,254	158	Gut (21 DPH)	R-1	11,890	46		R-6	27,865	41
	R-2	57,958	89		R-2	86,382	53	Gut (50 DPH)	R-1	14,749	37
	R-3	21,949	109		R-3	24,917	157		R-2	63,826	38
	R-4	19,455	74		R-4	20,857	111		R-3	32,812	116
	R-5	19,622	46		R-5	15,509	100		R-4	83,470	46
Gut (7 DPH)	R-1	14,621	68		R-6	13,246	75		R-5	1,01,927	41
	R-2	14,086	71	Gut (28 DPH)	R-1	57,355	66		R-6	66,131	41
	R-3	13,533	60		R-2	12,911	17	Gut (60 DPH)	R-1	85,356	82
	R-4	14,186	69		R-3	84,743	71		R-2	92,063	54
	R-5	12,895	87		R-4	43,597	126		R-3	1,16,025	31
	R-6	16,608	62		R-5	18,711	178		R-4	1,11,053	77
Gut (10 DPH)	R-1	16,608	69		R-6	24,378	47		R-5	87,647	55
	R-2	16,212	40	Gut (35 DPH)	R-1	73,890	98	Gut (75 DPH)	R-1	40,260	43
	R-3	12,765	50		R-2	11,0453	84		R-2	35,428	45
	R-4	13,137	122		R-3	94,864	94		R-3	26,988	27
	R-5	16,768	95		R-4	82,922	45		R-4	1,03,348	35
	R-6	12,569	78		R-5	14,767	67		R-5	64,926	30

**Sample type**	**Repliaction**	**Filtered sequences**	**No. of ASVs**	**Sample type**	**Repliaction**	**Filtered sequences**	**No. of ASVs**	**Sample type**	**Repliaction**	**Filtered sequences**	**No. of ASVs**
Gut (90 DPH)	R-1	73,386	50	FW (3 DPH)	R-3	1,13,439	153	FW (35 DPH)	R-2	78,653	149
	R-2	77,930	48	FW (7 DPH)	R-1	1,45,644	229		R-3	1,39,468	157
	R-3	40,013	37		R-2	1,05,222	225	FW (42 DPH)	R-1	1,26,121	267
	R-4	15,587	67		R-3	1,31,625	213		R-2	1,49,414	288
	R-5	31,086	22	FW (10 DPH)	R-1	1,41,764	215		R-3	1,12,082	277
	R-6	1,00,479	74		R-2	1,30,096	227	FW (50 DPH)	R-1	93,049	271
Feed (FW)	Feed (35 DPH)	75,551	73		R-3	94,656	215		R-2	1,25,451	259
Feed (FW)	Feed (42 DPH)	95,451	77	FW (14 DPH)	R-1	94,109	251		R-3	1,33,387	266
Feed (FW)	Feed (50 DPH)	41,688	64		R-2	72,664	248	FW (60 DPH)	R-1	1,34,773	268
Feed (FW)	Feed (60 DPH)	1,12,991	78		R-3	91,602	262		R-2	1,22,088	258
Feed (FW)	Feed (75 DPH)	87,961	73	FW (21 DPH)	R-1	90,084	189		R-3	91,240	244
Feed (FW)	Feed (90 DPH)	81,452	84		R-2	72,352	181	FW (75 DPH)	R-1	96,019	215
FW (Embryo)	R-1	89,621	138		R-3	97,955	201		R-2	1,01,654	209
	R-2	1,09,935	203	FW (28 DPH)	R-1	77,915	253		R-3	89,357	197
	R-3	1,09,288	154		R-2	43,952	190	FW (90 DPH)	R-1	1,09,170	267
FW (3 DPH)	R-1	91,703	142		R-3	1,34,032	282		R-2	77,433	218
	R-2	1,39712	151	FW (35 DPH)	R-1	78,830	159		R-3	1,31,299	263
Feed (FW)	Feed (FW, Stock)	1,57,133	74	Feed (SW)	Feed (SW, 7D)	1,15,022	63	Feed (FW)	Feed (FW, 21D)	1,55,599	59
Feed (SW)	Feed (SW, 1D)	1,28,282	71	Feed (FW)	Feed (FW, 14D)	1,06,737	62	Feed (SW)	Feed (SW, 21D)	1,59,494	68
Feed (FW)	Feed (FW, 7D)	1,25,665	62	Feed (SW)	Feed (SW, 14D)	1,37,651	55	Feed (FW)	Feed (FW, 28D)	1,43,484	56

Sample type	Repliaction	Filtered sequences	No. of ASVs	Sample type	Repliaction	Filtered sequences	No. of ASVs	Sample type	Repliaction	Filtered sequences	No. of ASVs
Gut (FW, Stock)	R-1	11,890	69	Gut (14D FW)	R-1	24,659	60	Gut (28D FW)	R-5	24,527	75
	R-2	83,894	69		R-2	26194	51	Gut (28D SW)	R-1	25,789	54
	R-3	53,027	61		R-3	194078	52		R-2	36,110	73
	R-4	44,814	59		R-4	103523	53		R-3	30,928	75
	R-5	64,822	60		R-5	14138	54		R-4	25,987	82
	R-6	28,696	46		R-6	25789	55		R-5	25,145	73
Gut (1D FW, Control)	R-1	71,958	81	Gut (14D SW)	R-1	70,284	76		R-6	25,789	75
	R-2	82,851	80		R-2	1,19,378	73	Water (FW, Stock)	R-1	1,27,700	110
	R-3	73,018	102		R-3	33,786	78		R-2	1,28,459	124
	R-4	52,763	72		R-4	32,015	61		R-3	1,43,989	109
	R-5	65,322	81		R-5	16,311	86	Water (FW, Control)	R-1	1,49,298	185
Gut (1D SW)	R-1	36085	65		R-6	1,10,672	64		R-2	1,52,225	183
	R-2	83225	73	Gut (21D FW)	R-1	79,137	73		R-3	1,45,130	215
	R-3	4318	71		R-2	90,793	69	Water (SW, Control)	R-1	1,43,197	245
	R-4	14914	65		R-3	1,31,723	75		R-2	1,08,869	239
	R-5	16194	66		R-4	39,063	59	Water (FW, 1D)	R-1	1,39,270	93
Gut (7D FW)	R-1	96,995	66		R-5	37,644	62		R-2	1,42,143	102
	R-2	93,984	46		R-6	11,717	73		R-3	1,25,122	103
	R-3	86,989	62	Gut (21D SW)	R-1	1,56,231	75	Water (SW, 1D)	R-1	1,71,176	163
	R-4	22,235	60		R-2	19,354	79		R-2	1,72,754	161
	R-5	51,168	45		R-3	90,021	72	Water (FW, 7D)	R-1	1,10,833	149
	R-6	24,736	48		R-4	1,30,217	76		R-2	1,70,291	157
Gut (7D SW)	R-1	56,858	60		R-5	2,29,181	73		R-3	1,66,417	149
	R-2	95,895	86		R-6	91,544	75	Water (SW, 7D)	R-1	2,02,878	264
	R-3	35,412	65	Gut (28D FW)	R-1	27,652	73		R-2	1,16,031	254
	R-4	50,383	66		R-2	67,710	75		R-3	1,51,987	238
	R-5	48,667	61		R-3	16,748	56	Water (FW, 14D)	R-1	1,18,158	154
	R-6	64,879	69		R-4	19,581	59		R-2	1,07,665	168

Sample type	Repliaction	Filtered sequences	No. of ASVs	Sample type	Repliaction	Filtered sequences	No. of ASVs	Sample type	Repliaction	Filtered sequences	No. of ASVs
Water (FW, 14D)	R-3	98,328	155	Water (FW, 21D)	R-1	1,51,786	166	Water (SW, 21D)	R-1	1,40,441	210
Water (SW, 14D)	R-1	1,00,179	215		R-2	1,67,316	173		R-2	1,40,922	203
	R-2	1,11,893	204		R-3	1,84,544	156		R-3	1,45,757	213
	R-3	58,468	189	Water (SW, 28D)	R-1	1,94,738	243				
Water (FW, 28D)	R-1	1,46,470	214		R-2	1,67,083	241				
	R-2	1,49,584	231		R-3	1,76,854	234				
	R-3	1,34,483	206								
